# Historical dynamics and current environmental effects explain the spatial distribution of species richness patterns of New World monkeys

**DOI:** 10.7717/peerj.3850

**Published:** 2017-09-26

**Authors:** Paulo Vallejos-Garrido, Reinaldo Rivera, Oscar Inostroza-Michael, Enrique Rodríguez-Serrano, Cristián E. Hernández

**Affiliations:** Laboratorio de Ecología Evolutiva y Filoinformática, Departamento de Zoología, Facultad de Ciencias Naturales y Oceanográficas, Universidad de Concepción, Concepción, Chile

**Keywords:** Amazonian Basin, Energy-richness hypothesis, Environmental constraints, Macroecology, Neotropic, Nested matrix, Hotspot

## Abstract

**Background:**

Why biodiversity is not uniformly distributed on the Earth is a major research question of biogeography. One of the most striking patterns of disparity in species distribution are the biodiversity hotspots, which generally do not fit with the distribution of relevant components of the Neotropical biota. In this study, we assess the proximal causes of the species-richness pattern of one of the most conspicuous groups of Neotropical mammals, the New World monkeys the Platyrrhini. We test two complementary hypotheses: (1) there is a historical source-sink dynamic (addressed using macroevolutionary and macroecological approaches); (2) the large number of species in the Amazon basin is due to the constraints imposed by environmental variables occurring outside this area.

**Methods:**

We first characterize spatial patterns of species richness and biodiversity hotspots using a new, objective protocol based on probabilities. Then we evaluate the source-sink hypothesis using BioGeoBEARS analysis and nestedness analysis of species richness patterns. Complementarily, to measure how often different species pairs appear in the same sites, we used null models to estimate the checkerboard score index (*C*-score). Finally, we evaluate the relationship between several climatic variables and species richness through ordinary least squares (OLS) and spatial autoregressive (SAR) models, and the potential environmental constraints on the pattern.

**Results:**

We found one significant cluster of high values for species richness in the Amazon basin. Most dispersal events occurred from the Amazonian subregion to other Neotropical areas. Temperature (T), discrepancy (BR), and NODF indexes show a significant nesting in the matrix ordered by species richness and available energy. The *C*-score observed was significantly smaller than the null expectation for all sites in the Neotropics where there are records of platyrrhine species. Ten climatic variables comprised the best-fitting model that explains species richness. OLS and SAR models show that this set of variables explains 69.9% and 64.2% of species richness, respectively. Potential of evapotranspiration is the most important variable within this model, showing a linear positive relationship with species richness, and clear lower and upper limits to the species richness distribution.

**Discussion:**

We suggest that New World monkeys historically migrated from their biodiversity hotspot (energetically optimal areas for most platyrrine species) to adjacent, energetically suboptimal areas, and that the different dispersal abilities of these species, the lack of competitive interactions at a macroecological scale, and environmental constraints (i.e., energy availability, seasonality) are key elements which explain the non-uniform pattern of species richness for this clade.

## Introduction

Why biodiversity is not uniformly distributed on the Earth is a major research question in biogeography and macroecology (Gotelli, 2000). One of the most striking patterns of this disparity in species’ distributions is the observation of biodiversity hotspots (Myers et al., 2000; Myers, 2003). The term biodiversity hotspot is now most commonly used with reference to regions of high species richness (Harcourt, 2000). However, earlier references considered biodiversity hotspots to be areas with a high proportion of endemic species facing significant threats due to habitat loss (Myers, 1988) or a particularly species-rich area with rare or threatened species, or some combination of these attributes (Reid, 1998). However, no definition has been based on a spatial-probabilistic approach to evaluate the statistical significance of the hotspot, which could allow us to recognize non-uniform distributions of biodiversity.

**Figure 1:**
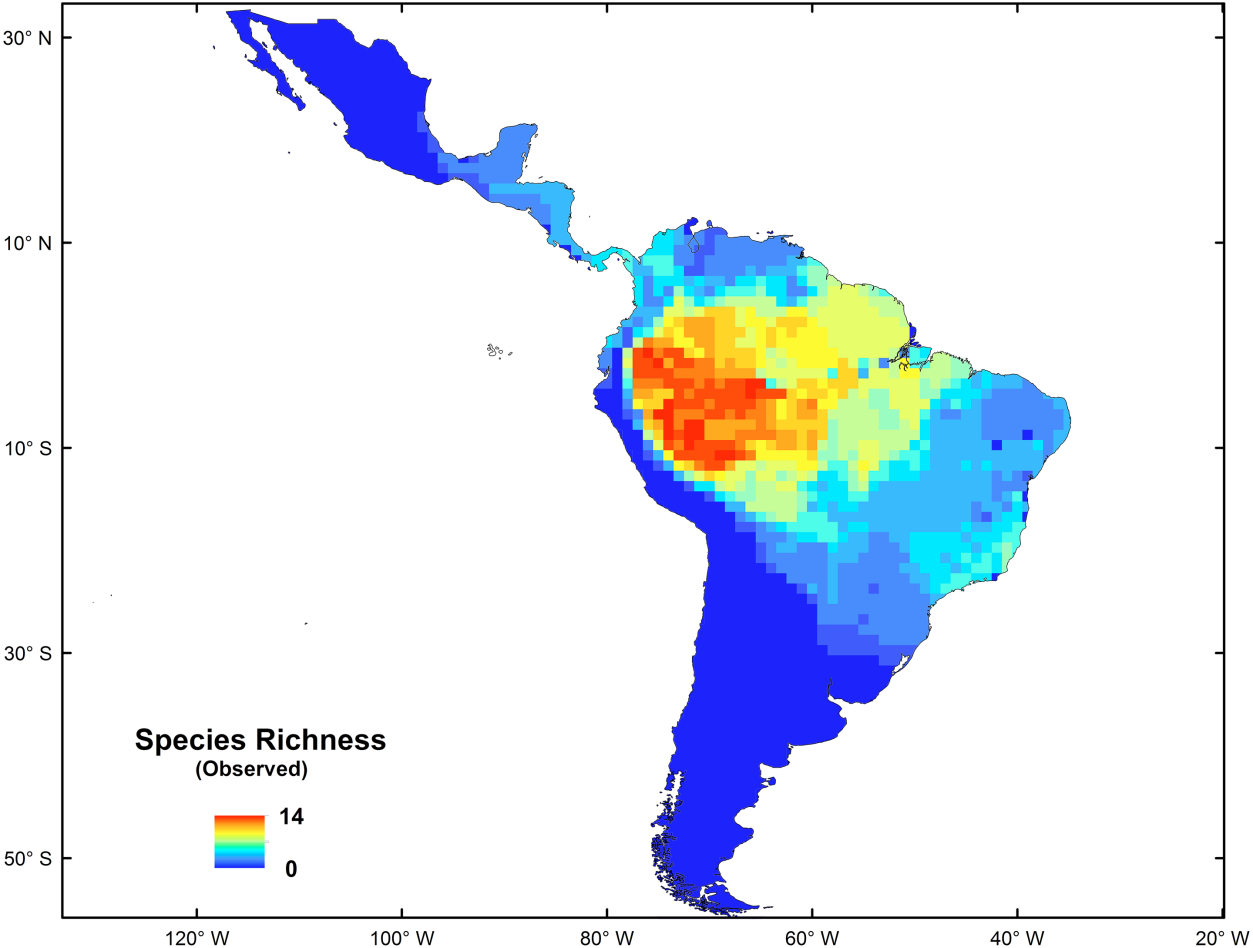
Spatial distribution of species richness for New World monkeys in the Neotropics.

The platyrrhine primates, also called New World monkeys, are one of the more conspicuous and representative elements of modern Neotropic mammalian fauna. This is a diverse group, with 150 living species grouped in 20 genera (Rylands, Mittermeier & Silva, 2012; Mittermeier, Rylands & Wilson, 2013), which occur in forested habitats from the northern border of the tropical forest in southern Mexico (20°N) to subtropical regions of northern Argentina and southern Brazil (30°S) (Peres & Janson, 1999; Goldani, Carvalho & Bicca-Marques, 2006; Kay et al., 2012); they have colonized almost every niche available in the Neotropics (Lynch-Alfaro et al., 2015). This group currently shows a longitudinal increase of species richness from east to west, and a latitudinal decrease from the Amazonian Basin to southern South America and to Central America (See Fig. 1) (Voss & Emmons, 1996). However, this distribution pattern is not completely consistent with the biodiversity hotspots presented by Myers et al. (2000) for the Neotropics. The origins and evolution of such high Platyrrhine diversity in the Amazonian Forest remain poorly understood (Tejedor, 2013; Aristide et al., 2015; Bond et al., 2015; Jameson Kiesling et al., 2015; Lynch-Alfaro et al., 2015), but some hypotheses have been proposed (e.g., the riverine hypothesis; Peres, Patton & Da Silva, 1996; Boubli et al., 2015; Lecompte et al., 2017). Recently, Jameson Kiesling et al. (2015) proposed that the origin and initial diversification of the most recent common ancestor of platyrrine primates occurred in the Amazon, giving rise to existing species by local diversification in the last 15 Myr, suggesting that many genus- and species-level divergences within each of the Aotidae, Atelidae, Callitrichidae, Cebidae, and Pithecidae families occurred first in the Amazon and subsequently spread to the Atlantic Rainforest, Cerrado, Caatinga and Central Grasslands, coinciding with the development and expansion of the Amazonian Rainforest (Pulliam & Danielson, 1991; Lynch Alfaro et al., 2012; Buckner et al., 2015; Morales-Jimenez, Disotell & Fiore, 2015).

However, while evolutionary studies have explicitly addressed the diversification processes of this Amazonian species pool (Aristide et al., 2015; Boubli et al., 2015; Buckner et al., 2015; Jameson Kiesling et al., 2015; Morales-Jimenez, Disotell & Fiore, 2015), little effort has been made to explain the current spatial pattern of species richness (Kay et al., 1997; Peres & Janson, 1999; Stevenson, 2001; Mercado & Wallace, 2010). In this sense, Ulrich & Gotelli (2007) proposed that research efforts should focus on the underlying mechanisms of species richness patterns, given that these can result from: (1) interspecific interactions (Ulrich, Jabot & Gotelli, 2017), and (2) physical-climatic variables (Stevenson, 2001). For the first mechanism, there is scant evidence that predation has a direct effect on reducing platyrrhine richness. However, changes in population densities and some local extinctions can occur in areas with a strong human pressure from the hunting of large primates (Peres, 1990; Stanford, 1995). Also, competition for resources among primate species explains the distribution of a few parapatric species (Terborgh, 1986). In fact, Fleming (1979) evaluated the resource overlap and food levels used by Neotropical frugivorous primates, concluding that there are no significant levels of interspecific competition in this group. Consequences of species interactions have been documented from local to regional scales, but they have not been evaluated in the Neotropics as a whole (Fleming, Breitwisch & Whitesides, 1987; Peres, 1997). With regard to the second mechanism, many studies have proposed that the energy available in the environment and its covariation with climate is a generality, and one of the most important variables for determining the spatial structure of vertebrate communities, that may restrict the number of coexisting species (Hutchinson & MacArthur, 1959; Wright, 1983; Hawkins et al., 2003; Currie et al., 2004; Lomolino et al., 2006). In the Neotropics, the maximum energy available is found in the Amazon basin and adjacent areas where the species richness of the New World monkeys also peaks (i.e., energetically optimal habitat for most of platyrrhine species) (Kay et al., 1997; Rahbek & Graves, 2001). Thus, species with greater tolerance ranges and dispersal abilities can survive in areas with less energy (i.e., energetically suboptimal habitat), and less tolerant species become extinct or specialize in lower and upper limits of climatic and energetic ranges (Pulliam & Danielson, 1991). Given that platyrrhines have diversified in the Amazon basin, with posterior dispersal to Neotropical areas following a pattern similar to the energetic gradient, we propose a historical source–sink dynamic as a first joint macroecological-macroevolutionary hypothesis to explain the current pattern of species richness for New World monkeys. These historical source–sink dynamics can be represented by: (1) a macroevolutionary dispersal model from the “center of origin” or “cradle of diversity” (i.e., the region where the species pool is generated by local speciation, but not immigration, such as the Amazon basin) to a macroevolutionary sink area that obtains taxa only through immigration (Goldberg, Lancaster & Ree, 2011); and (2) a macroecological nested pattern of species richness correlated with available energy and associated with a higher concentration of species than expected by chance (Pulliam, 1988; Almeida-Neto, Guimaares & Lewinsohn, 2007). In the latter hypothesis, given that in the Amazon basin many primate species distributions overlap, producing a maximum species richness area, we expect that areas outside the Amazon basin should be a nested subset of the species composition of larger assemblages. Also, as a complementary, non-historical hypothesis we propose that the large number of species in the Amazon basin is due to the constraints on the Platyrrhini imposed by climatic conditions (available energy and correlated variables) occurring outside this area.

In this study we evaluate the proximal causes of the pattern of species richness of New World monkeys in the Neotropics. First, we characterize spatial patterns of species richness and biodiversity hotspots using a new, objective protocol based on probabilities. Second, we conduct several exploratory analyses to identify proxies of energy availability and its correlated climatic variables that are associated with species richness. Evaluate two main predictions: (1) that there is a historical source–sink dynamic (i.e., macroevolutionary source–sink model, nested pattern of species richness, and pattern of species co-occurrence greater than expected by chance); and (2) that there is a positive and significant correlation between species richness and available energy, which, imposes marked constraints on platyrrhine richness outside of the biodiversity hotspot.

## Materials and Methods

### Data collection: climatic variables and distribution maps

We used the distribution maps of 125 species of New World monkeys obtained from “NatureServe Digital Distribution Maps of the Mammals of the Western Hemisphere Version 3.0” (Patterson et al., 2007). Range distribution map data provide a wide geographical coverage of information, often at a 1° cell resolution (roughly 100 × 100 km), which is fine enough to provide detail about diversity variations, and coarse enough to not compromise the reliability of the derived biodiversity measures (Carnicer et al., 2007; Hortal et al., 2008). Information about species richness was obtained by the overlap of these maps of species’ distributions (Hurlbert & Jetz, 2007; Hortal, 2008; Da Silva, Provete & Hawkins, 2016). Neotropical environmental data were obtained from several databases, including: proxies of available energy (http://sedac.ciesin.columbia.edu/es/hanpp.html; Imhoff et al., 2004); potential of evapotranspiration (PET; Trabucco & Zomer, 2009); and actual evapotranspiration (AET; Ahn & Tateishi, 1994). River density was obtained by calculating the density of bodies of water, using a model of the hydrological network for South America available on HydroSHEDS portal (Hydrological data and maps based on Shuttle Elevation Derivatives at multiple scales: http://www.hydrosheds.org) at a resolution of 30 arc-seconds (Lehner, Verdin & Jarvis, 2008). The river density estimations were performed using the Spatial Analyzer module, subroutine line density of ArcGis 10.2 (ESRI, 2016). The topographic position index (TPI), a proxy of topographic heterogeneity, was calculated from a Digital Elevation Model (http://srtm.csi.cgiar.org/) using ArcGis 10.2 (ESRI, 2016). Altitude (m.a.s.l.) and altitude range (m) data were obtained from the Wordclim portal (Hijmans et al., 2005) at a resolution of 30 arc-seconds. Also, we obtained 19 contemporary bioclimatic variables that describe the variation in monthly, quarterly and annual measures of precipitation and temperature from the WorldClim database (Hijmans et al., 2005), and these variables were analyzed with the goal of exploring other possible factors associated with the biogeographic pattern of primates. Given that cells can span both sides of rivers, and thus could overestimate the observed species richness within each cell, we reanalyzed the data by removing all adjacent cells to the main Amazon river channels. This procedure was repeated for all predictors of platyrrhine richness: temperature, precipitation, productivity, etc. Management and exploration of environmental data, as well as the calculation of Neotropical species richness were done in ArcGIS 10.2 software (ESRI, 2016). To compare how species richness patterns change or remain the same over different spatial resolutions (0.5°, 0.6°, 0.7°, 0.8°, 0.9°, and 1°), we used the similarity measurement, Kappa (Zachwatowicz, 2011). Kappa indicates the spatial distribution based on concordance between two maps, where a value of 1 indicates total agreement between the two maps of species richness and −1 indicates that the two maps are completely different. The analyses were performed using Map Comparison Kit 3.2.3 software (Visser & De Nijs, 2006) (see results in Supplemental Information 1). The final analysis was done at 1° resolution because: (1) finer grid analyses yielded similar patterns (Supplemental Information 1); (2) at finer grid sizes range maps overestimate the area of occupancy for individual species and mischaracterize spatial patterns of species richness, which can result in spurious biodiversity hotspots (Hurlbert & White, 2005; Hurlbert & Jetz, 2007); and (3) the main environmental variables in the models are transversal to spatial scales (see below).

### Hotspot determination

Several studies have been performed to identify biodiversity hotspots (e.g., Bonn, Rodrigues & Gaston, 2002; Orme et al., 2005). However, these studies rely on multiple criteria, which attempt to establish areas where species richness is above an arbitrary cut-off value of richness (often between 1% and 5%; Orme et al., 2005). Consequently, none of the previous studies have determined biodiversity hotspots based on spatial-statistical criteria to describe the patterns of biodiversity, which is fundamental for analyzing raw biodiversity data in order to extract information and make justified decisions (see Webb & Copsey, 2011). With the aim of developing an objective protocol based on probabilities to detect biodiversity hotspots, we used an intuitive measure, which detects cells, or groups of cells (i.e., spatial clusters), containing greater species richness than expected by chance for a given taxon and study area. First, we determined if platyrrhine species richness in the Neotropics showed spatial autocorrelation, using Moran’s *I* statistic. If the results show positive and significant autocorrelation, there is clustering of high values of species richness. Alternatively, negative autocorrelation points to a scenario of greater dispersal than expected by chance. No evidence of autocorrelation indicates a random pattern. Second, we defined spatial hotspots using the Gi* statistics (Getis & Ord, 1992). Briefly, Gi* identifies spatial concentrations of an entity (attributes coded as presence-absence; in this case species richness per cell) or areas that contain high values. To establish a statistically significant hotspot, an entity must have high values and be surrounded by other cells with high values. Accordingly, the local sum for an entity and its neighbors is compared proportionally with the sum of all entities. If the local sum is extremely different from the random expectation, a significant *Z* score is assigned. Significant values of *Z* > 0 provide evidence for significant hotspots whereas values of *Z* < 0 provide evidence for groups of entities that have lower values than expected by chance. The statistical determination of hotspots was performed in ArcGIS 10.2 software (ESRI, 2016).

### Evaluation of the source-sink hypothesis

#### Macroevolutionary approach

To evaluate a source–sink dynamic in a historical context, we estimated the ancestral distribution for the pavorder Platyrrhini and the number of dispersal events between the areas, using the BioGeoBEARS package (Matzke, 2013) implemented in the R language (R Development Core Team, 2008). BioGeoBEARS allows for the estimation of ancestral geographic ranges on dated phylogeny, comparing several models of range evolution. We used the DEC model (Ree & Smith, 2008) with two free parameters: “d” (dispersal rate) and “e” (extinction rate), and a fixed cladogenetic model (cladogenetic event allowed: vicariance, sympatric-subset speciation, and sympatric range-copying). We also used a DEC model with an extra parameter, “j”, which represents the founder-event speciation, where the new species “jumps” to a range outside of the ancestral range (DEC + j model). The comparison of these two models was performed using Akaike Information Criterion (AIC). To perform this analysis, we used a scheme similar to the classification of the areas used by Jameson Kiesling et al. (2015), but since their map is not available in GIS format, we used the biogeographic classification proposed by Morrone (2001): (A) Central Andes Subregion, (B) Amazonian Subregion, (C) Chacoan Subregion, and (D) Atlantic Forest Subregion. This analysis was performed based on the time-calibrated maximum clade credibility (MCC) tree published by Aristide et al. (2015). This time-calibrated tree was estimated using 15 nuclear genes and four mitochondrial genes selected from Perelman et al. (2011) based on 78 species, being, to our knowledge, the most complete time-calibrated phylogenetic tree for this clade.

Based on the parameters of the best supported biogeographic model (DEC or DEC + j), we performed Biogeographic Stochastic Mapping (BSM), as implemented in the BioGeoBEARS R package (Matzke, 2013). The BSM generates simulated biogeographic histories, including the time and allocation of all biogeographic events (e.g., within-area speciation, vicariance and dispersal events) occurring along the branches. The event frequencies were estimated from the event counts of 1,000 BSM’s. The historical source–sink dynamic under a phylogenetic-based biogeographic analysis (i.e., DECs models and BSM) is supported if the main platyrrhine lineages originated in the Amazonian area, and the dispersal events were more frequent from the Amazon to the rest of the Neotropical regions. The R script is available in Supplemental Information 2.

#### Macroecological approach

We performed a nestedness analysis to evaluate our first hypothesis. Nested assemblages are evident when impoverished sites tend to be simple subsets of the richest ones, suggesting a highly ordered system in which colonization/extinction dynamics may be actively shaping species occurrences across sites (Cutler, 1998). Thus this approach corresponds to an indirect evaluation of a historical hypothesis, implicitly incorporating a temporal component for large spatial scales (e.g., Neotropics; Allen & Gillooly, 2006). For the nestedness analysis we considered only areas with species records, generating a grid of 300 cells of 2°for the Neotropics. Then we constructed a presence-absence species richness matrix, where the columns represent the 300 cells and the rows represent the species. This matrix was ordered according to the total sum of rows and columns, locating the most frequent species in the upper rows and the richest sites on the left side of the matrix. Also, aiming to evaluate if available energy had the same spatial distribution as platyrrhine species richness, we constructed a matrix for AET values, which was ordered by productivity following the same logic as the species richness matrix. The T, discrepancy (BR; Brualdi & Sanderson, 1999) and the NODF (Almeida-Neto et al., 2008) indexes were used to estimate the degree of nestedness. For the latter index, in addition to estimating the overall nestedness of the matrix, it also allows independent calculation of the degree of nesting of columns (i.e., species composition) and rows (i.e., species incidence). The statistical significance of these indexes was evaluated by the generation of a null model using a Monte Carlo algorithm, contrasting the observed values with a null probability distribution. In our case, we used a null model with fixed rows and equiprobable columns, where the total number of rows is maintained but the column totals vary randomly (Patterson & Atmar, 1986; Gotelli, 2000). This null model maintains the frequency of occurrence of species and allows species richness to vary equiprobably between sites (Valencia-Pacheco et al., 2011). We selected this model considering that all sites could be occupied by species, with no effect or bias derived from geography or species interactions. Previous studies using simulations have shown that altering the sum of the rows may generate vulnerability to type I statistical error (Gotelli, 2000). To generate the frequency distribution of null data, a total of 10,000 iterations were performed. All analyses were performed in the NODF software (Ulrich & Gotelli, 2013).

### Co-occurrence analysis

Aiming to measure how often different species pairs appear in the same sites, we used null models to estimate the checkerboard score index (*C*-score). This is considered one of the best indexes to determine species co-occurrence patterns (Gotelli, 2000). An observed *C*-score was calculated and compared to indexes derived from 50,000 null matrices (randomly assembled matrices). The model employed to generate null matrices was based on the row sums fixed and equiprobable columns algorithm, which has low type I error, good power to detect non-random patterns, and is recommended when the data matrix has many zeros and sampled communities are homogeneous (Gotelli, 2000; Lopez et al., 2013). This algorithm considers that the mean number of occurrences for each species in the null communities is equal to the observed data set, and the sites have equal probability to be represented in the null communities. Since the matrix was narrowed to sites with species records (previous section), and the New World monkeys have colonized almost every niche available in their distribution range (i.e., a continuum), the row sums fixed and equiprobable columns is an appropriate algorithm to represent the natural pattern. The *C*-score in a competitively structured community should be significantly greater than expected by chance. Because raw *C*-score values vary depending on co-occurrences observed at each site, we calculated a standardized effect size (SES) for the matrix in order to compare results across sites. SES’s were calculated as: (observed *C*-score—mean of simulated *C*-scores)/standard deviation of simulated *C*-scores. If values are positive then there are less co-occurrences than expected by chance, whereas if they are negative then there are more co-occurrences. The first case is indicative of competition, while more co-occurrence is a sign of facilitation (Lopez et al., 2013; Vaz et al., 2015). Assuming a normal distribution of deviations, approximately 95% of the SES values should fall between −2 and 2 if co-occurrences are not different from that expected by chance. This co-occurrence analysis was performed with EcoSim7.5 software (Gotelli & Entsminger, 2006).

### Relationships between climatic variables and species richness

We generated a matrix in which each cell has the following information: longitude, latitude, species richness, the nineteen climatic variables (Bio1-Bio19) available in WorldClim, PET, AET, topographic position index (TPI), river densities, altitude, and altitude range. Multiple linear regressions were performed using the “stepwise forward” method, and the main variables that contributed to the species richness of New World monkeys were selected using AICc and *R*^2^ statistics, in SAM software (Rangel, Diniz-Filho & Bini, 2010). The association between environmental factors and species richness was explored by two approaches: ordinary least squares (OLS) and the spatial autoregressive (SAR) model; the latter incorporates spatial autocorrelation in the model structure (Dormann et al., 2007; Kissling & Carl, 2008). The best model to describe this relationship was selected by AICc and *R*^2^ statistics.

The interaction between environmental factors and species richness at very large geographic scales can shed light on some environmental constraints to the distribution of species richness (in this case energy constraints; Hawkins et al., 2003). Thus, we estimated the Pearson correlation coefficient (*ρ*) as measure of the linear relationship between species richness and environmental variables, and we evaluated the upper and/or lower limit in the “species richness–climatic variable” space, determining the linear regression of the highest (99th quantile) and lowest (1st quantile) significant quantiles using the “Quantreg” package (Koenker, 2013) in the R software (R Development Core Team, 2008). This approach establishes the significance of the slope (with the null hypothesis of slope = 0) using the rank score test for quantile regression, and evaluates the probability (*p*) of a Chi-square distribution using a bootstrap approach (for this analysis we used 10,000 randomizations). The R script is available in Supplemental Information 2.

## Results

### Hotspot determination

**Figure 2:**
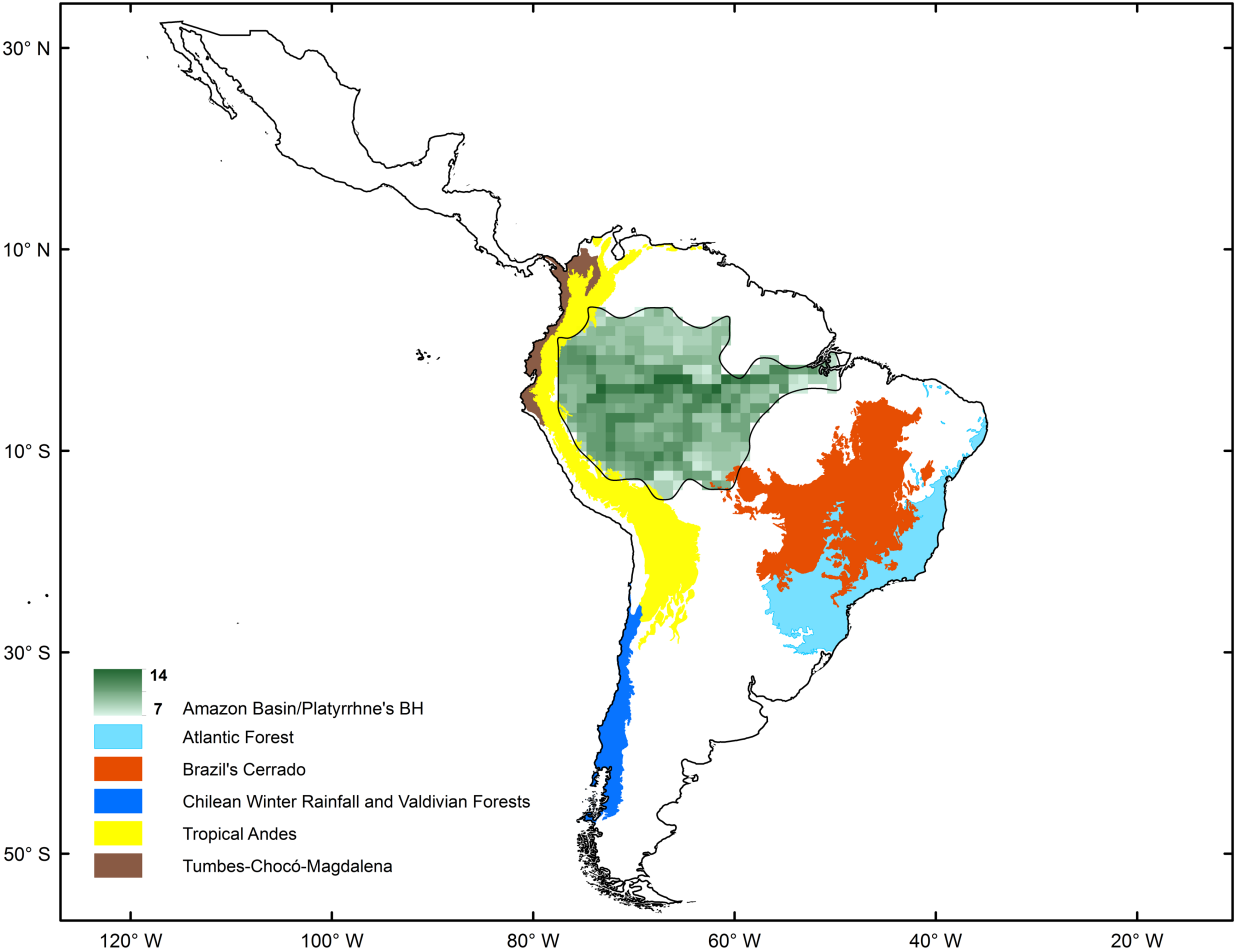
Comparison of biodiversity hotspots for New World monkeys determined by statistical analysis (green) and the Neotropical Biodiversity Hotspots proposed by Myers et al. (2000).

The spatial statistics analyses proposed to identify hotspots for New World monkeys indicated that there is an area where species richness peaks. Values of species richness were positively autocorrelated (Moran’s Index: obs = 0.88; *Z* score = 40.7, *p* < 0.05); the Getis-Ord Gi* statistic indicated that there is one cluster of high values for species richness (*Z* = 24.73; *p* < 0.01) (Fig. 2). This hotspot is composed of 140 cells with higher species richness than expected by chance (Fig. 1). The hotspot follows the course of the Amazon River from the eastern Andean flanks to the Atlantic Ocean (Fig. 2).

### Evaluation of the source-sink hypothesis and co-occurrence analyses

**Figure 3:**
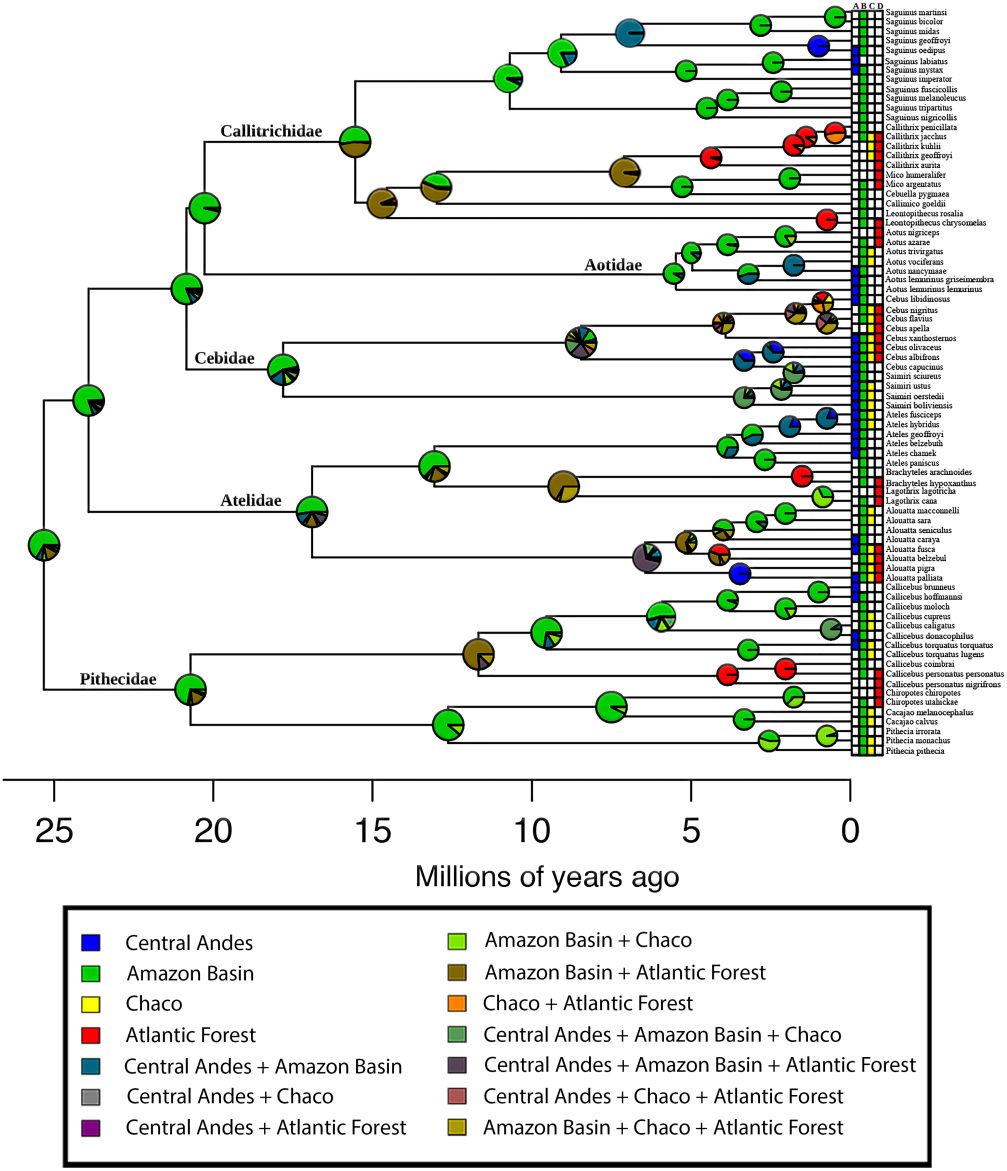
Biogeographical analysis of New World Monkeys using BioGeoBEARS. The four biogeographical areas are: (A) Central Andes (in blue); (B) Amazonian Subregion (in green); (C) Chacoan Subregion (in yellow); and (D) Atlantic Forest (in red). Outgroups are not shown. Pie charts at nodes indicate support for respective areas. Tips are labelled with present-day species distributions. The secondary colors indicate range combinations of the tip ranges.

The AIC model selection on the biogeographic model implemented in BioGeoBEARS indicated that a DEC model is the best-supported (Table 1). Based on this model, the estimation of ancestral areas for the extant diversity of the Platyrrhini placed the most probable ancestral area in the Amazonian biogeographic subregion (Fig. 3). Furthermore, the main lineages (families) belonging to the clade, also probably originated in the Amazonian subregion (Fig. 3). The dispersal summary extracted from the 1,000 BSM’s maps reveals that most of the dispersal events occurred from the Amazonian subregion to the other biogeographic areas considered in this work (Table 2). The results of the ancestral area estimation and number of dispersal events suggest that Amazonia is the center of origin for this clade, from which the species colonized the rest of the Neotropical region. These results also support the historical source–sink dynamic for the origin of platyrrhine biogeographic pattern.

**Table 1:** Biogeographic Stochastic Mapping (BSM). Models are dispersal–extinction–cladogenesis (DEC), dispersal–extinction–cladogenesis allowing for founder-event speciation (DEC + j).

Models	ML	DF	*d*	*e*	j	AIC
DEC	**−150.2872457**	**2**	**0.026677605**	**2.001E–09**	**0**	**304.5744914**
DEC + j	−149.2852047	3	0.024613064	1E–12	0.012945479	304.5704095

**Notes.**

ML Maximum-likelihood DF degrees of freedom *d* rate of dispersal *e* rate of extinction *j* relative probability of founder-event speciation at cladogenesis AIC Akaike’s information criterion AIC_c_ corrected Akaike’s information criterion

Our results also show a significant matrix nestedness, with values for the T and BR indexes smaller than expected by chance, and the value of the NODF index greater than expected by chance (*P* < 0.0001, Table 3). In addition, we found independent significant nesting between rows (i.e., incidence of species) and columns (i.e., species composition) for species richness, with NODF values greater than expected by chance (*P* < 0.0001, Table 3). Similar results were found for the species richness matrix ordered by available energy in the Neotropics (measured by AET), which also showed significant nesting with index values lower than expected by chance for the T, BR and NODF columns, and a larger row NODF index than expected by chance (*P* < 0.0001, Table 3). The *C*-score observed for the data using the analysis of co-occurrence was significantly smaller than the average of the 50,000 simulations for all sites in the Neotropics where there are records of platyrrhine species. The Standardized Effect Size was negative and significant (*C*-score = 331.73 < simulated mean *C*-score = 351.39; variance = 1.43; SES = −16.46).

### Relationships between climatic variables and species richness

**Figure 4:**
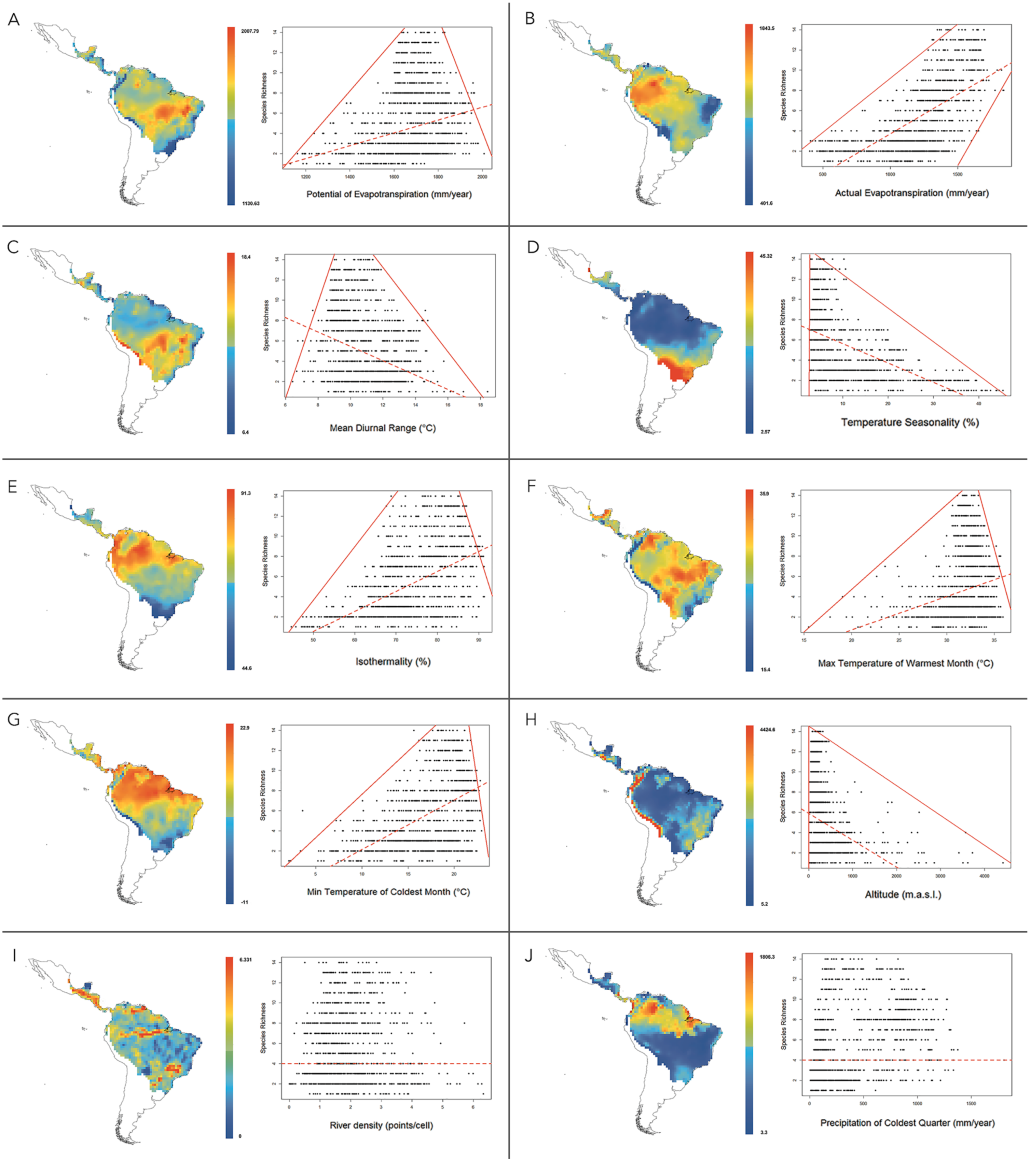
Maps for variation of climatic variables in the distribution range of New World monkeys. We present relationships between species richness and different climatic variables for the 1,229 sites in the Neotropics; red lines are 01th quantile, 99th quantile and OLS reggresion. (A) Potential Evapotranspiration; (B) Actual Evapotranspiration; (C) Mean Diurnal Temperature Range; (D) Temperature Seasonality; (E) Isothermality; (F) Max Temperature of Warmest Month; (G) Min Temperature of Coldest Month; (H) Altitude; (I) River density; (J) Precipitation of Coldest Quarter.

At 1°resolution, the analysis of multiple linear regressions showed that the best-fitting model (AICc = 19821.037 and *R*^2^ = 0.699) contained ten climatic variables (Table 4). The OLS and SAR models showed that this set of climatic variables explained 69.9% and 64.2% of platyrrhine richness, respectively. Multiple regression analysis revealed that PET was the most important environmental variable that predicts the species richness of New World monkeys. According to correlation and regression tests, PET showed a significant positive relationship with species richness (*ρ* = 0.23; *p* ≤ 0.01), with a significant upper and lower limit (*p* ≤ 0.01) that shows a constraint on the number of species, given the low available environmental energy, and the maximum number of species exhibited by a geographic cell tended to increase at medium values of PET (Fig. 4A). Similarly, a positive relationship was found for AET (*ρ* = 0.66; *p* < 0.01). In this case, the lower limit does not allow the presence of few species in areas with higher available energy (above 1500 mm evapotranspiration), i.e., the minimum and maximum number of species found in a geographic cell tended to increase with AET (Fig. 4B). Mean temperature diurnal range (ρ =  − 0.28; *p* < 0.01) and temperature seasonality (*ρ* =  − 0.54; *p* < 0.01) showed a significant negative relationship with species richness, describing a triangular polygon that constrain species number to sites with high values of these variables, *i.e.*, platyrrhines are absent in sites where the mean diurnal temperature range exceeded 18 °C (Fig. 4C). Only low temperature seasonality (<10%) allows high species richness (Fig. 4D). Other variables that describe temperature variation in the Neotropics behaved differently; isothermality (ρ = 0.52; *p* < 0.01), the minimum temperature of the coldest month (ρ = 0.51; *p* < 0.01), and the maximum temperature of the warmest month (ρ = 0.22; *p* < 0.01), also describe a triangular polygon, where sites with higher values of these variables allow the co-occurrence of 1 to 14 species (Figs. 4E–4G). Altitude also shows a negative and significant relationship (ρ =  − 0.34; *p* < 0.01), where the maximum species number is found from 0 to 1,000 m.a.s.l. with a marked decrease at higher altitudes (Fig. 4H). Precipitation in the coldest quarter shows a significant positive relationship (*ρ* = 0.38; *p* < 0.01), but does not show a clear geometric constrains on species richness. Finally, river density does not show a significant relationship (*ρ* =  − 0.015; *p* = 0.59), and neither a geometric pattern, despite influencing the multiple regression model (Figs. 4I and 4J). PET, AET and Temperature Seasonality, are transversal variables at all spatial scales (i.e., 0.5°, 0.6°, 0.7°, 0.8°, 0.9°, 1°) and explain the major portion of species’ richness, while river density is absent in only one spatial scale, but their contribution to species’ richness is very low and independently is null (see Tables 4 and 5).

**Table 2:** The dispersal summary extracted from 1,000 BSM’s maps.

	Caribbean subregion + North Andes	Amazonian subregion	Chacoan subregion	Atlantic forest
Caribbean subregion + North Andes	0	2.257	1.043	0.527
Amazonian subregion	8.455	0	9.11	4.894
Chacoan subregion	0.996	2.268	0	2.063
Atlantic forest	0.492	1.397	4.508	0

**Table 3:** Nestedness pattern based on the species richness matrix of the New World monkeys ordered by (A) species number and by (B) energy availability. The table shows the T, BR, and NODF indices for the group, and the degree of nestedness for columns and rows, independently, using the NODF index.

A	T index	BR index	NODF index	NODFc index	NODFr index
Metric obs	4.20^*^	1,037^*^	14.05^*^	15.29^*^	6.86^*^
Metric sim	5.69	1097.40	15.08	16.77	5.29
(CI)	(5.24–6.15)	(1,083–1,112)	(14.49–15.70)	(16.10–17.47)	(5.04–5.56)
**B**	**T index**	**BR index**	**NODF index**	**NODFc index**	**NODFr index**
Metric obs	8.82^*^	1,602^*^	19.76^*^	20.91^*^	13.12^*^
Metric sim	12.56	1734.87	18.21	19.65	9.88
(CI)	(11.93–13.19)	(1,718–1,751)	(17.72–18.71)	(19.11–20.20)	(9.59–10.18)

**Notes.**

CI, 95% Confidence Interval.

* *P* < 0.0001.

**Table 4:** OLS and SAR models for the relationship between species richness and environmental predictors of the best model given a respective spatial scale.

Species richness scale	Best model (Variables ordered by partial cofficient in absolute value)	OLS *R*^2^	SAR *R*^2^	AICc
0.5	PET–Bio3–Bio7–Bio5–Bio1–Bio10–Bio16–Bio13–AET–Bio4–Bio6–Bio17–Bio19–Range altitude–Altitude–River density	**0.689**	**0.677**	**19821.037**
0.6	Range altitude–River density–Altitude–AET–Bio3–Bio4–Bio2–Bio19–Bio17	**0.527**	**0.49**	**15231.003**
0.7	PET–Bio2–Bio1–Bio5–Bio3–AET–Bio4–Bio17–Range altitude–Altitude	**0.681**	**0.676**	**10178.837**
0.8	PET–Bio2–Bio1–AET–Bio6–Bio17–Bio4–Bio19 – Range altitude–Altitude–River density	**0.664**	**0.677**	**7796.842**
0.9	PET–Bio6–AET–Bio4–Bio19–Altitude–River density	**0.673**	**0.679**	**6210.117**
1.0	PET–Bio2–Bio5–Bio3–Bio6–AET–Bio4–Bio19–Altitude–River density	**0.699**	**0.642**	**5157.685**

**Notes.**

PET Potential of evapotranspiration AET Actual evotranspiration Bio1 Annual Mean Temperature Bio2 Mean Diurnal Range Bio3 Isothermality Bio4 Temperature Seasonality Bio5 Max Temperature of Warmest Month Bio6 Min Temperature of Coldest Month Bio7 Annual Temperature Range Bio10 Mean Temperature of Warmest Quarter Bio13 Precipitation of Wettest Month Bio15 Precipitation Seasonality Bio16 Precipitation of Wettest Quarter Bio17 Precipitation of Warmest Quarter Bio19 Precipitation of Coldest Quarter AICc corrected Akaike Information Criterion

In red, environmental variables transversal to all scales.

**Table 5:** OLS and SAR models for the relationship between species richness and environmental predictors at 1° of resolution

Variable	OLS coeff.	AICc	OLS *R*^2^	SAR coeff.	AICc	SAR *R*^2^	Std. coeff.	Std. error	*P* value
		**4951.127**	**0.699**		**5157.685**	**0.642**			
Constant	−115.208			−110.103			0	12.501	<0.001
Potential of evapotranspiration	160.319			130.869			1.386	13.154	<0.001
Mean diurnal range	−67.725			−57.367			−1.049	6.057	<0.001
Max temperature of warmest month	−73.524			−53.485			−0.503	13.257	<0.001
Isothermality	−30.822			−22.858			−0.372	4.331	<0.001
Min temperature of coldest month	−13.391			−10.958			−0.408	1.679	<0.001
Actual evapotranspiration	7.379			6.8			0.224	0.928	<0.001
Temperature seasonality	−4.581			−3.811			−0.33	0.655	<0.001
Precipitation of coldest quarter	−1.954			−1.541			−0.221	0.235	<0.001
Altitude	1.592			1.415			0.163	0.318	<0.001
River density	−0.89			−0.779			−0.059	0.227	<0.001

## Discussion

The origin and diversification of the extant platyrrhine species occurred in the Amazonian forest during the Late Oligocene and Early Miocene (Jameson Kiesling et al., 2015). These species have extended and contracted their ranges in parallel with the expansion and fragmentation of the Amazonian tropical forests, which provided a rich and complex ecosystem, where the diversification of the major platyrrhine clades occurred in sympatry (Hoorn & Wesselingh, 2010; Jameson Kiesling et al., 2015). Our results based on macroecological (i.e., nestedness analysis and co-occurrence) and macroevolutionary (i.e., BioGeoBEARS) approaches support this historical proposal according to: (1) the significant species-richness cluster associated with the Amazonian Basin (Fig. 2); (2) strong nested and co-occurrence patterns in species richness; and (3) the historical origin of this clade in the Amazonian region, associated with posterior high frequency dispersal out of this area, which constitutes the source of diversity for the rest of the Neotropical region. Thus, the current pattern shows us that impoverished areas (outside the biodiversity hotspot) tend to be simple subsets of the richest ones, suggesting a highly ordered system in which colonization/extinction dynamics may be actively shaping species occurrences across areas. In this case, the dynamic is dominated by dispersal events from the Amazon basin. In fact, based on nesting analysis and null models of species occurrence, the existence of a marked nested pattern in species richness distribution for the Platyrrhini suggests that the distribution of species is not determined by chance. Significant nesting also occurred independently in rows and columns within the species matrix of the Platyrrhini, indicating that incidence of species and species composition for each site have been due to colonization ability and extinction susceptibility (Ulrich & Gotelli, 2013). This is especially valid for non-island systems such as the Neotropics (Mendez, 2004). Interestingly, strong support for this comes from the scarce but important fossil record found in areas which platyrrhine species do not inhabit today (Hoffstetter, 1969; Flynn et al., 1995; Takai et al., 2000; Tejedor, 2013). For example, †*Chilecebus carrascoensis* (20 Mya) was found in the Mediterranean of Chile (35°S; Flynn et al., 1995); which is far from the current distribution of the Platyrrhini (Fig. 1). This fossil species lived in this region when the Andes had a mean elevation of 1,000 m, allowing the tropical forest to reach southern latitudes. After the most recent Andean uplift during the Late Miocene, the tropical forest was replaced by the semiarid Mediterranean sclerophyll forest which does not support any extant primate species (Fig. 1; Hinojosa, Armesto & Villagrán, 2006). Thus, historical emigration from the Amazon basin, associated with a gradient in available energy, seems to be the dominant process that generated the nested pattern in species richness. Since habitats are usually connected by migration, some generalist species that originated in productive habitats colonized the adjacent energetically suboptimal range, expanding the spatial distributions. The expansion started during the mid-Miocene when Amazonia underwent considerable changes beginning with a northward continental drift, resulting in increased temperature and the presence of a humid tropical setting (Pardo-Casas & Molnar, 1987; Jaramillo, Rueda & Mora, 2006). By the beginning of the Late Miocene, Amazonia looked much as it does today (Hoorn & Wesselingh, 2010), and nearly all of the extant platyrrhine genera had diversified, some in sympatry (changing the species composition in the source area), others expanding their ranges (changing the species composition in sink areas), and others migrating to subproductive areas in the Neotropics (changing the species composition in source and sink areas). Species that have limited dispersal can often be absent in an energetically suboptimal habitat, due to the difficulty of reaching these areas (e.g., *Saguinus imperator*, *Alouatta seniculus*). In contrast, active dispersal of species from the Amazon basin could maintain large sink populations, and such dispersal could be evolutionarily stable, developing a nested structure of species richness. Therefore, we emphasize the importance of dispersal ability and extinction processes in generating the source–sink dynamics and the observed richness pattern, especially considering that some species can be very vagile and have a wide range of distribution that covers areas with low and high available energy (Ayres & Clutton-Brock, 1992; Jablonski, Roy & Valentine, 2006; Lecompte et al., 2017). Also, our results show that in the Neotropics there is higher species co-occurrence than expected by chance, and the significant nestedness score indicates that interspecific competition may be not a factor that determines the nature of the species richness pattern. Traditionally, many authors have interpreted this positive, non-random association (aggregation) in terms of habitat filtering and facilitation (e.g., Götzenberger et al., 2012; Vaz et al., 2015). However, Ulrich, Jabot & Gotelli (2017), using birth-death community models, identified a trade-off between the type of competitive interactions and the degree of dispersal. Intraspecific competitive interactions can generate species aggregation, and segregation may arise from dispersal limitation alone; thus, some reported effects of competition on the geometry of species occurrences might require reassessment. In our case, the high co-occurrence appears to be related to the high dispersal rate of this group, and the lack of competitive interactions at a macroecological scale. Recently, Araújo & Rozenfeld (2014) based on extensive model simulations support the view that the spatial signature of negative interactions is sensitive to scale, i.e., exclusion by competitors at local scales of resolution tends not manifest at coarser scales (Greig-Smith, 1979; Woodward, 1987; Gotelli, Graves & Rahbek, 2010). They also demonstrate that interactions involving positive dependencies between species, such as mutualism (+∕ +) and commensalism (+∕0), are more likely to be manifested across different scales of resolution.

On the other hand, energy availability for primate species mainly takes the form of food availability (Kay et al., 1997; Peres & Janson, 1999; Stevenson, 2001). The Amazon basin is the Neotropical area with most energy flowing into a community system, and thus a site with high food availability. However, beyond the high available energy, constant temperatures and the correlated variables in Amazon basin also allow for the coexistence of a high number of sympatric species (Fig. 4). The variation of these variables throughout the Neotropics is the main determinant of the low number of species and the simple composition of species in areas outside of the biodiversity hotspot (i.e., Chaco, Atlantic Forest, Central Andes), with increasing extinction susceptibility towards the observed environmental constraints (Fig. 4). For example, if the annual precipitation is <1,000 mm/year, only 5 sympatric species can be found in a given area of 100 km^2^. A similar relationship was found between available environmental energy and primate species richness. Both increase with rainfall up to a maximum of 3,000 mm/year and then fall off at higher rainfall levels. In the biodiversity hotspot (Amazon basin) we found medium values of rainfall (between 2,300 and 3,000 mm/year; Fig. 4). Aiming to explain this relationship, we searched for causes of the decline of the AET and PET due to high levels of rainfall. Defler (2013) found that in the moist, tropical forest heavy rains (above 3,000 mm/year) leach soils of calcium, magnesium and potassium, causing a decrease in phosphorus availability and creating more acidic soil, all of which affect primary production and, by extension, platyrrhine richness (Myers, 1988; Pastor-Nieto & Williamson, 1998; Peres, 2008).

The platyrrhine biodiversity hotspot is characterized by high isothermality values between 76% and 92.16% (Fig. 4E) and the lowest temperature seasonality values (Fig. 4D). Strong seasonality negatively affects the density of frugivorous animals due to the seasonal food shortages. For example, in most temperate forests no fruits are available for several months of the year (Stevenson, 2001). Although the Atlantic coast of South America is not a temperate forest, this region has two well-defined seasons, (one dry and cold season and the one wet and warm season; Myers, 1988). This seasonality influences species richness because some species may not be able to tolerate the harsh dry season. Also, since in the Atlantic forest and Brazil’s Cerrado rainfall does not exceed 2,300 mm/year, the availability of environmental energy is considerably smaller than in the Amazon basin, so a maximum of eight sympatric species can coexist in 100 km^2^, including two endemic genera, *Brachyteles* and *Leontopithecus* (Rylands, Mittermeier & Silva, 2012). Related to the above variables, altitudinal gradient shows a marked decrease in species richness over 1,000 m.a.s.l. This pattern agrees with the traditional view of the relationship between diversity and elevation in tropical forests (Lieberman et al., 1996; Givnish, 1999), and the classic ecological theory that diversity and temperature decline with elevation due to the lowering of productivity and an increase in biotic interactions (Ruggiero, Lawton & Blackburn, 1998; Geise et al., 2004). For example, the most commonly found primates at high altitudes in Peru is the Atelidae, with species commonly occurring at elevations of 2,000 m.a.s.l. (Shanee et al., 2014). On the other hand, river density is a transversal explanatory variable at all spatial scales used in this study, but its contribution to the multiple regression model is very low, and independently, as a predictor variable its contribution is not significant. The geometric form in which Amazonian rivers configure the general species’ richness pattern is unclear. Ayres & Clutton-Brock (1992) first suggested that Amazonian rivers are effective barriers to primate species dispersal, largely related to their width, seasonal and annual stability, rate of flow, and the ability of species to cross these environmental barriers. However, despite the long-term stability of some of these watercourses, recent studies found no evidence that rivers acts as an effective barrier (Boubli et al., 2015; Lecompte et al., 2017). Thus, from a macroecological perspective, these lower and upper boundaries are the most important feature of the relationships between species richness and climatic variables, because they appear to represent a limit to the number of species for a given geographic area and a given combination of habitat properties. In this sense, we continue to provide evidence that that the limits of species ranges often match with combinations of climate variables (Woodward, 1987; Root, 1988), and that these limits shift through time in synchrony with changes in climate (Walther, Beißner & Burga, 2005; Hickling et al., 2006; Lenoir et al., 2010). It seems likely that species lying along these boundaries would have the highest probability of extinction as a result of any change in their environmental habitat conditions. At large scales, habitat selection and community processes are less important, and richness gradients and species replacement are more the outcome of the environmental limitations and dispersal ability of the group of species studied. Therefore, range map data can provide a sharp picture of these large-scale dynamics (Hortal, 2008).

In conclusion, we found evidence that New World monkeys historically migrated from their biodiversity hotspot (energetically optimal habitat) to adjacent, energetically suboptimal areas, and that the different dispersal abilities of these species, the lack of competitive interactions at a macroecological scale, and environmental constraints (i.e., energy availability, seasonality) are key elements which explain the non-uniform pattern of species richness for this clade.

##  Supplemental Information

10.7717/peerj.3850/supp-1 Supplemental Information 1 Resolution maps comparisons (A) Kappa values for the different resolutions of analysis of specific richness; (B) Biodiversity Hotspot delimitations to all spatial scales; (C) Biodiversity Hotspot defines using the Gi* statistics.

10.7717/peerj.3850/supp-2 Supplemental Information 2 R Scripts (A) Biogeographic stochastic mapping (BSM) in BioGeoBEARS package; (B) Ancestral area reconstruction in BioBeoBEARS package; (C) Quantile Regressions in Quantreg package.

## Additional Information and Declarations

### Data Availability

The following information was supplied regarding data availability: The distribution maps of 125 species of New World monkeys obtained from “NatureServe Digital Distribution Maps of the Mammals of the Western Hemisphere Version 3.0” (Patterson et al., 2007). Information about species richness was obtained by the overlap of these maps of species distribution (Hurlbert & Jetz, 2007). Proxies of energy availability http://sedac.ciesin.columbia.edu/es/hanpp.html; (Imhoff et al., 2004): potential of evapotranspiration (PET; Trabucco & Zomer, 2009) and actual evapotranspiration (AET; Ahn & Tateishi, 1994). River density was obtained by calculating the density of bodies of water, using a model of the hydrological network for South America available on HydroSHEDS portal (Hydrological data and maps based on Shuttle Elevation Derivatives at multiple scales: http://www.hydrosheds.org) at a resolution of 30 arc-seconds (Lehner, Verdin & Jarvis, 2008). Topographic position index (TPI), as a proxy of topographic heterogeneity, was calculated from a DEM http://srtm.csi.cgiar.org/ using ArcGis 10.2 (ESRI, 2016). Also, 19 contemporary bioclimatic variables that describe the variation in monthly, quarterly and annual measures of precipitation and temperature were obtained from the WorldClim database (Hijmans et al., 2005), and were analyzed with the goal of exploring other possible factors associated with this biogeographic pattern of primates. Management of environmental data and the calculation of Neotropical species richness were processed with the ArcGIS 10.2 software (ESRI, 2014) using grids of 1°× 1°(≈100 km^2^).
