# Supplementary material for: Historical dynamics and current environmental effects explain the spatial distribution of species richness patterns of New World monkeys
Source: PeerJ. 2017 Sep 26;5:e3850. doi: 10.7717/peerj.3850 (PMC5621511; doi:10.7717/peerj.3850)
Supplement: Supplemental Information 1 — (A) Kappa values for the different resolutions of analysis of specific richness; (B) Biodiversity Hotspot delimitations to all spatial scales; (C) Biodiversity Hotspot defines using the Gi* statistics. [file peerj-05-3850-s001.docx]

**SUPPLEMENTARY MATERIALS**

**Supplementary material 1: Resolution maps comparisons**

**A)**

According to the Kappa statistic, similarity in the species richness pattern, on the basis of 1° resolution, decreases as the resolution is increased, and does not exceed 30% similarity (Table 1). However, at 0.5° resolution, there is high similarity in the species richness pattern with 1° resolution (Table 1).

In a spatial context, the Kappa location statistic (i.e. spatial distribution of the different categories of richness in the study area) revealed that specific richness exhibits low similarity as the resolution is increased from 0.9 to 0.6 degrees, with the exception of the map at 0.5 ° (ca. 70% similarity). Conversely, according to the Kappa Histo statistic (i.e. total number of richness pixels), a greater similarity (KHisto >70%) was found between the maps of 0.9°, 0.8°, 0.7°, and 0.6° with 1°, reaching the highest similarity with the 0.5° resolution map (ca. 90% similarity; Table 1 and Figs. 1-5).

**Table 1**. Kappa values for the different resolutions of analysis of specific richness.

|  | **Kappa** | **Klocation** | **Khisto** |
| --- | --- | --- | --- |
| Resolution 1 vs 0.5 degree | 0.632 | 0.693 | 0.912 |
| Resolution 1 vs 0.6 degree | 0.344 | 0.435 | 0.79 |
| Resolution 1 vs 0.7 degree | 0.334 | 0.43 | 0.776 |
| Resolution 1 vs 0.8 degree | 0.334 | 0.424 | 0.787 |
| Resolution 1 vs 0.9 degree | 0.327 | 0.417 | 0.785 |

**
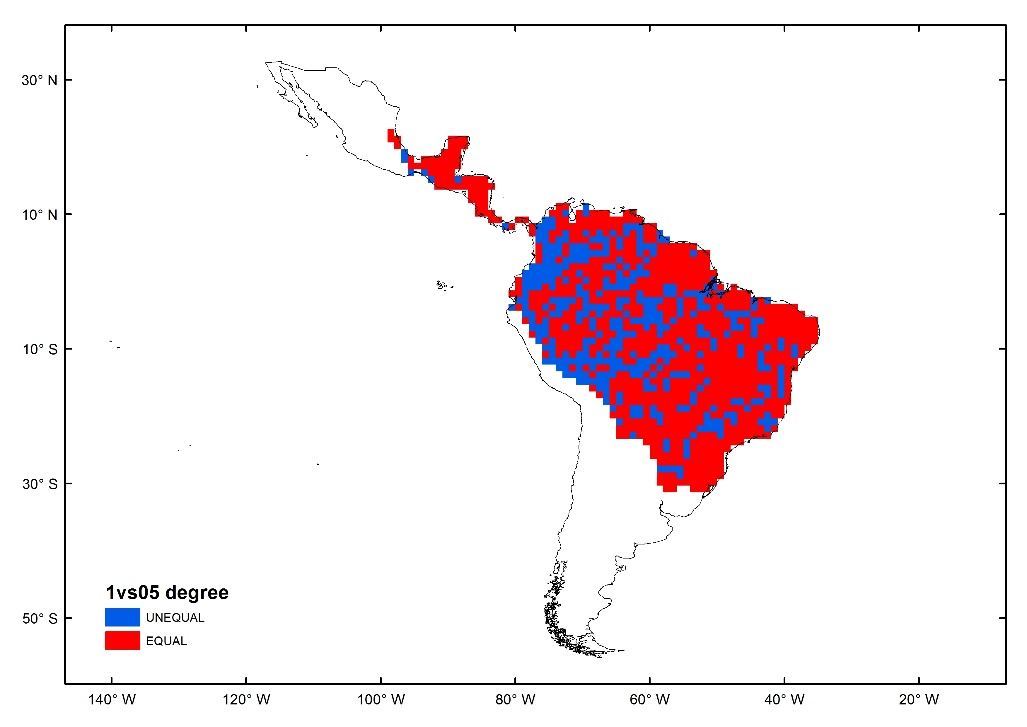
**

**Figure 1.** Comparison of species richness maps for 1° vs 0.5° resolutions. Red indicates equal cells, and blue indicates unequal cells.

**
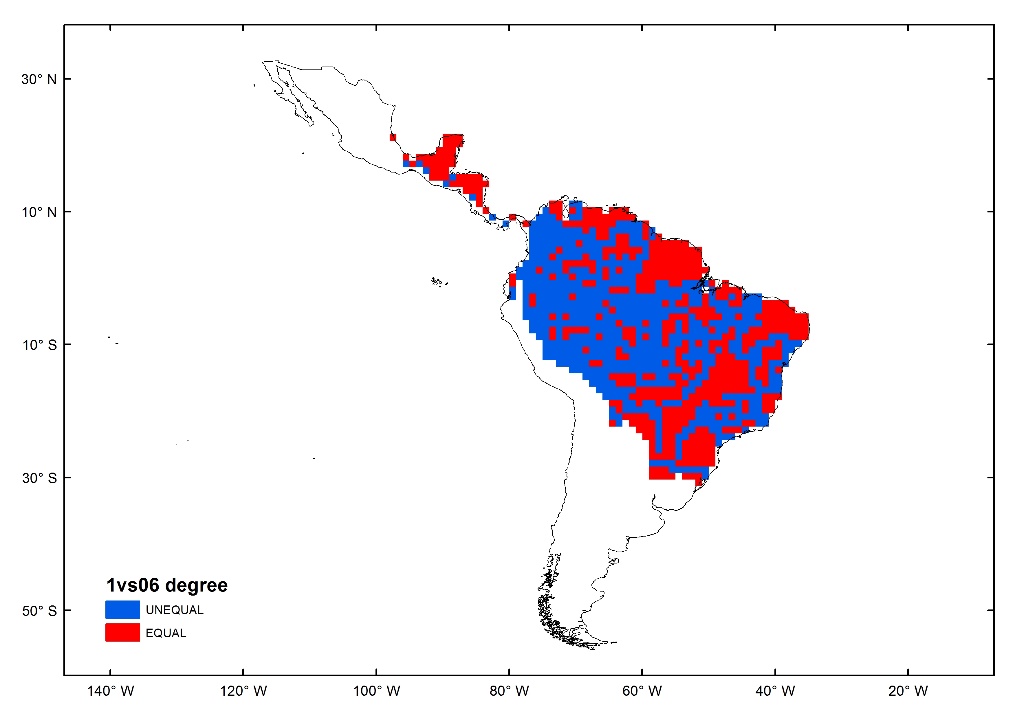
**

**Figure 2.** Comparison of species richness maps for 1° vs, 0.6° resolutions. Red indicates equal cells, and blue indicates unequal cells.

**
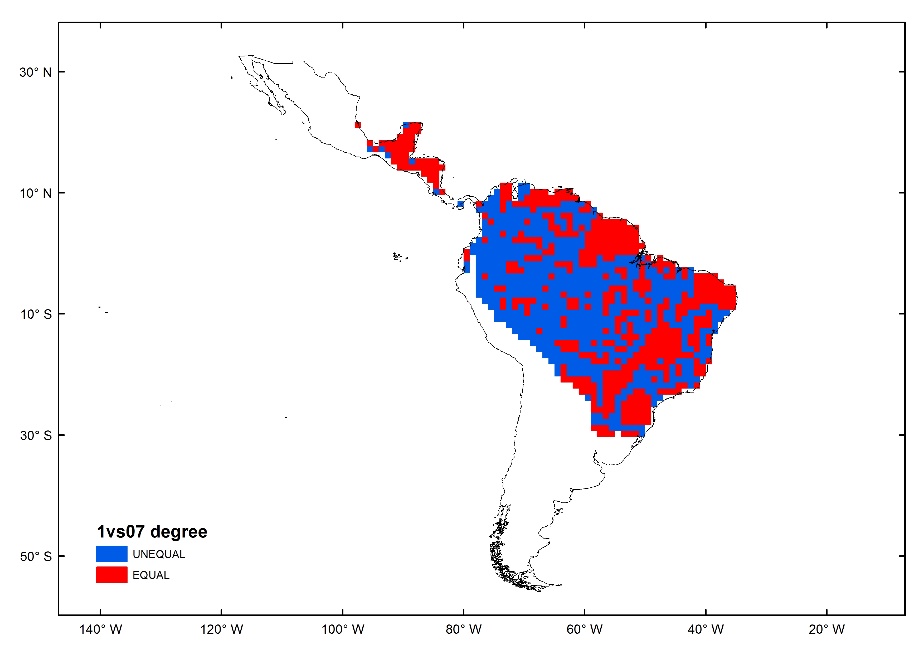
**

**Figure 3.** Comparison of species richness maps for 1°vs 0.7° resolutions. Red indicates equal cells, and blue indicates unequal cells.

**
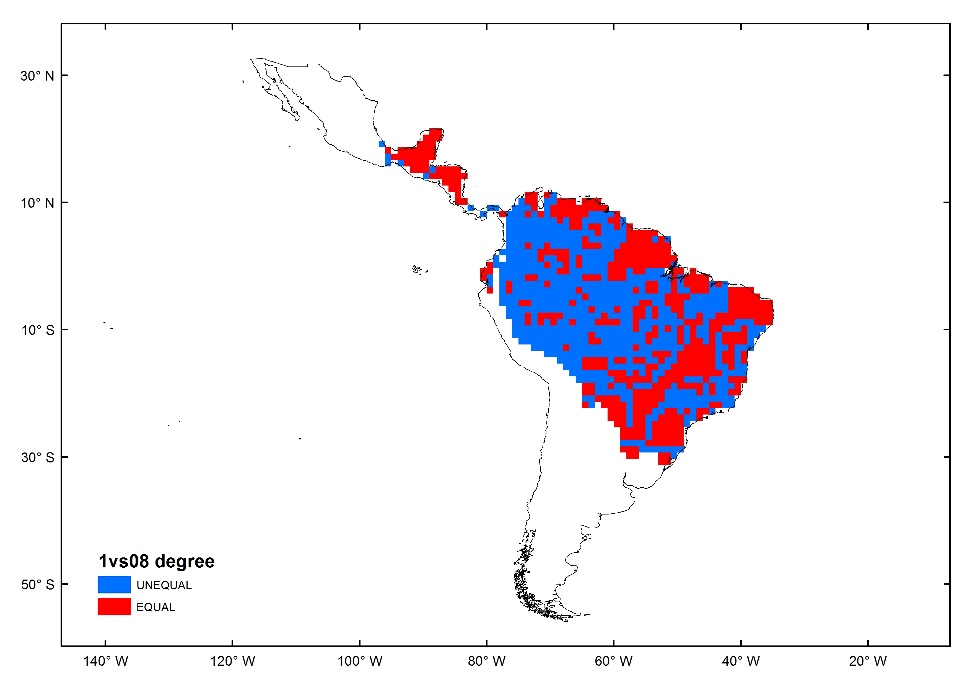
**

**Figure 4.** Comparison of species richness maps for 1°vs 0.8° resolutions. Red indicates equal cells, and blue indicates unequal cells.

**
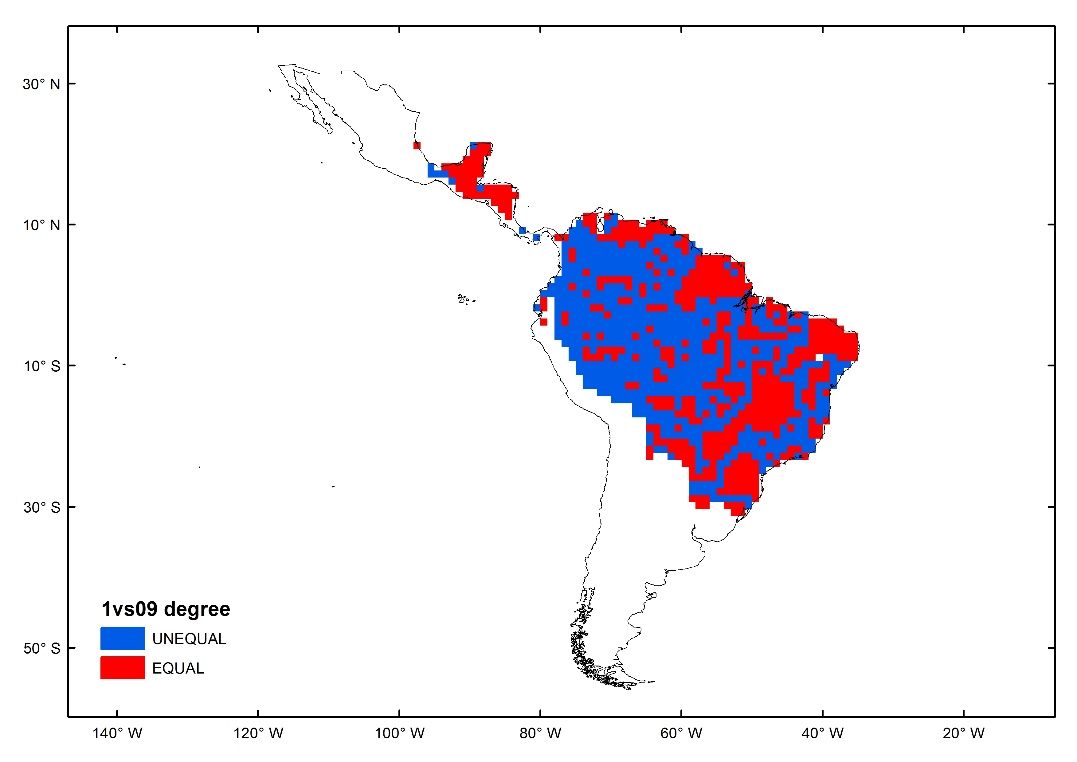
**

**Figure 5.** Comparison of species richness maps for 1° vs 09° resolutions. Red indicates equal cells, and blue indicates unequal cells.

**B)**

Gradients of species richness for the New World monkey Hotspot delimited by statistical analysis (rainbow color vector, where red is the maximum value) at different grid sizes **(Figures 6 to 10)**, and Neotropical Biodiversity Hotspots proposed by Myers et al. (2000).

**
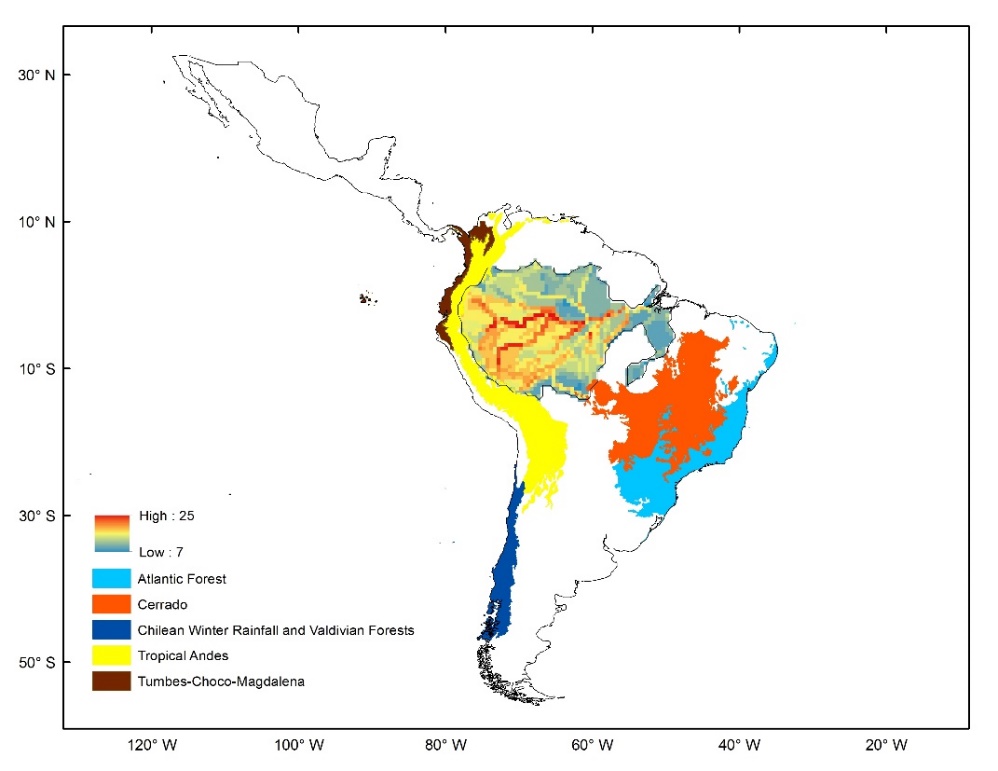
**

**Figure 6.** Biodiversity Hotspot delimitations to 0.5°

**
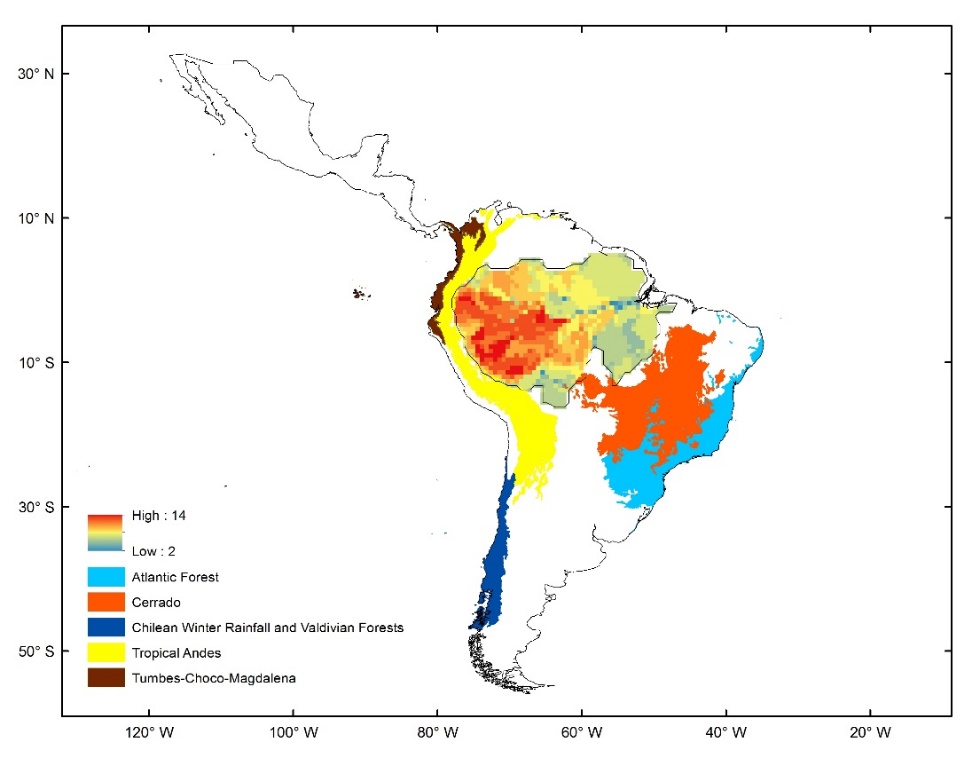
**

**Figure 7.** Biodiversity Hotspot delimitations to 0.6°

**
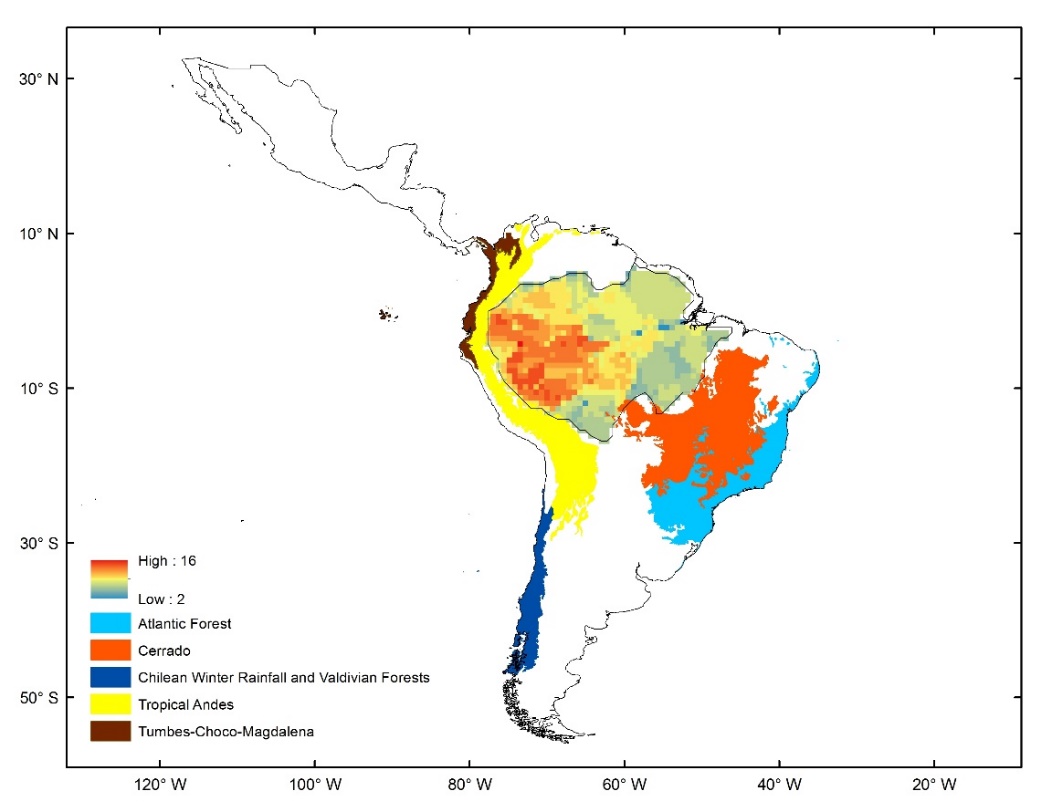
**

**Figure 8.** Biodiversity Hotspot delimitations to 0.7°

**
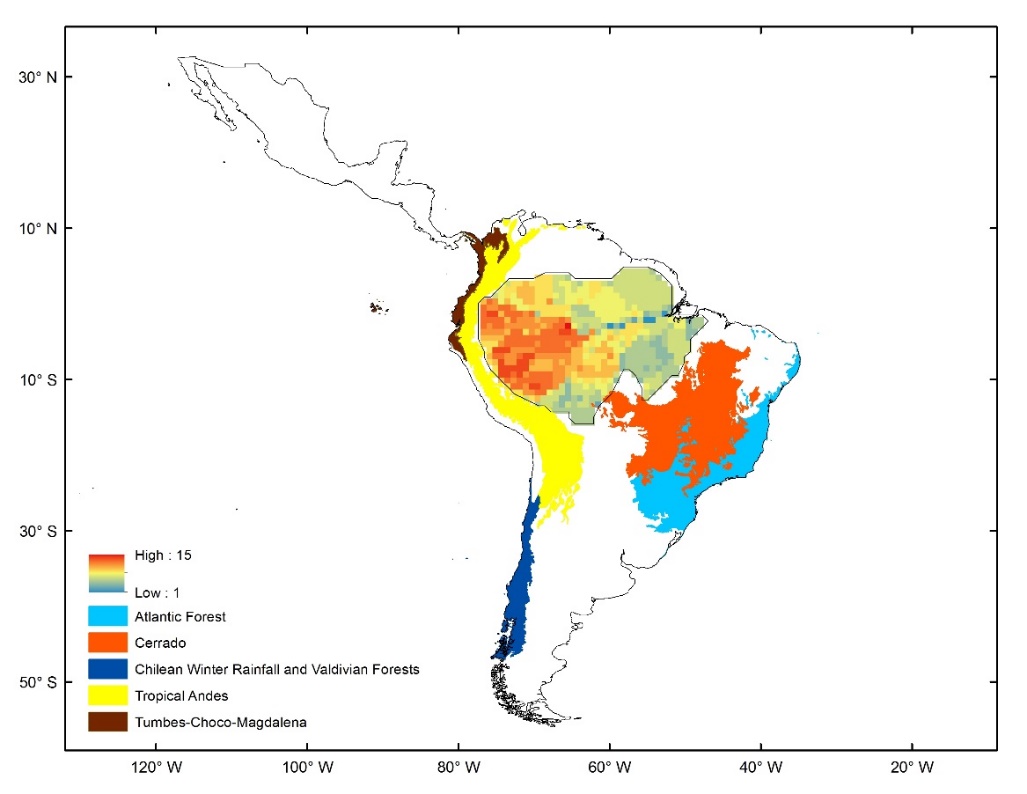
**

**Figure 9.** Biodiversity Hotspot delimitations to 0.8°

**
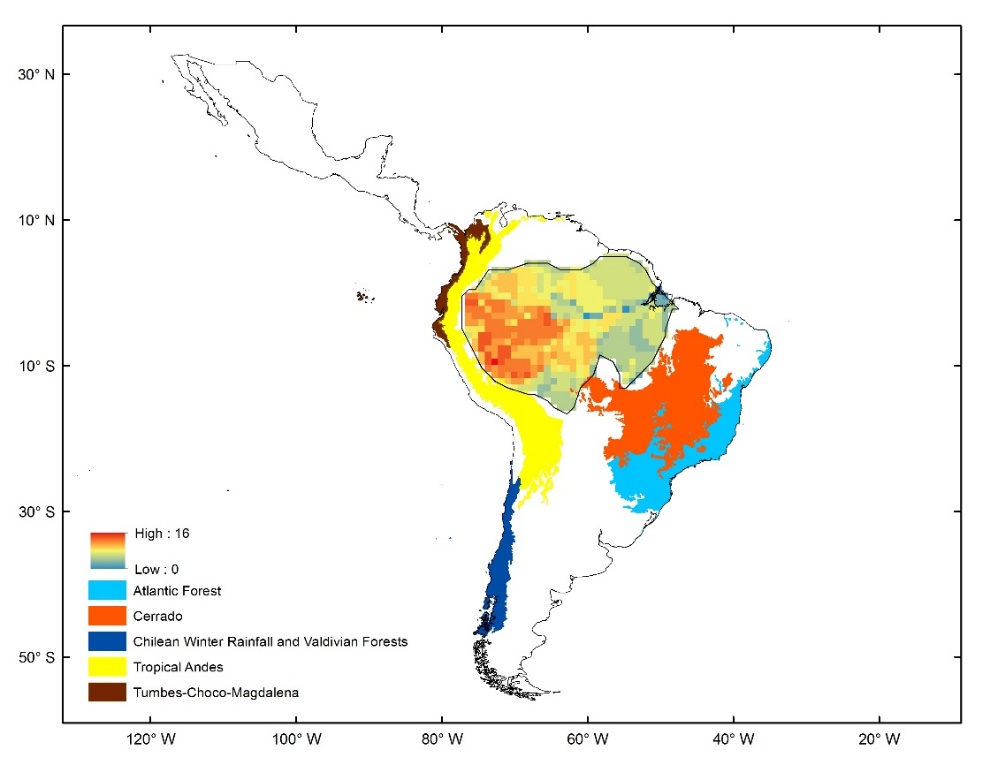
**

**Figure 10.** Biodiversity Hotspot delimitations to 0.9°

**C)**

Hotspot delimitation based on the Gi* statistics (Getis & Ord, 1992) at different grid sizes **(Figures 11 to 15):**

**
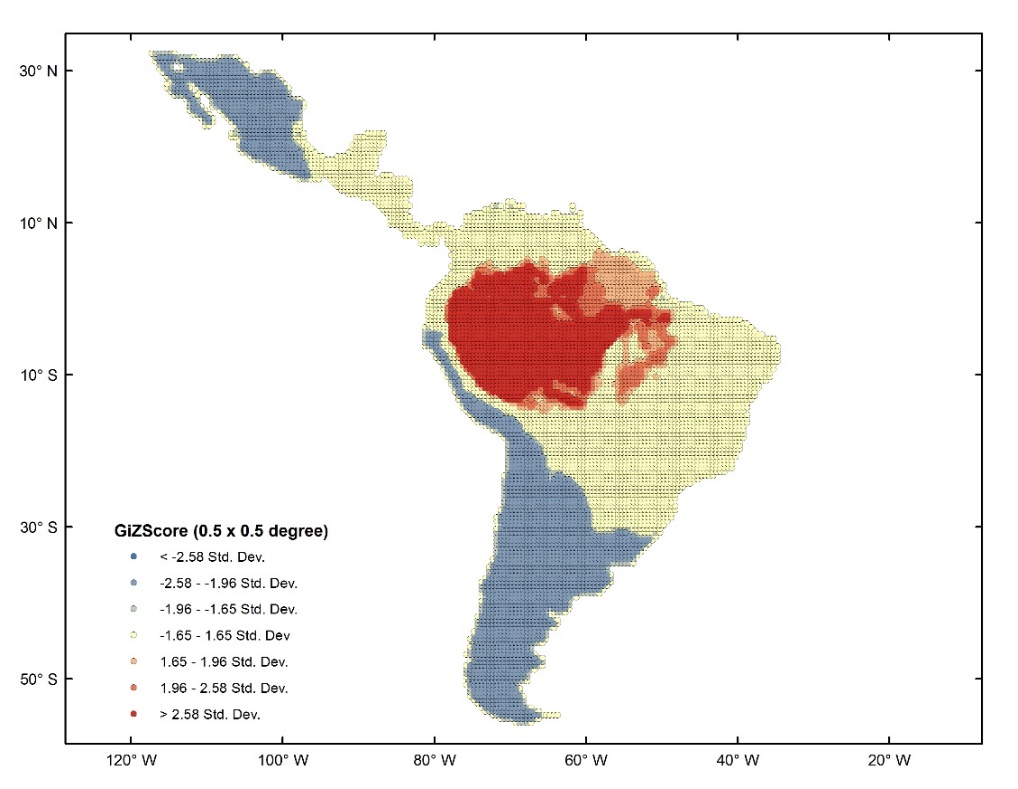
**

**Figure 11.** Biodiversity Hotspots defined using the Gi* statistics (Getis & Ord, 1992) to 0.5°.

**
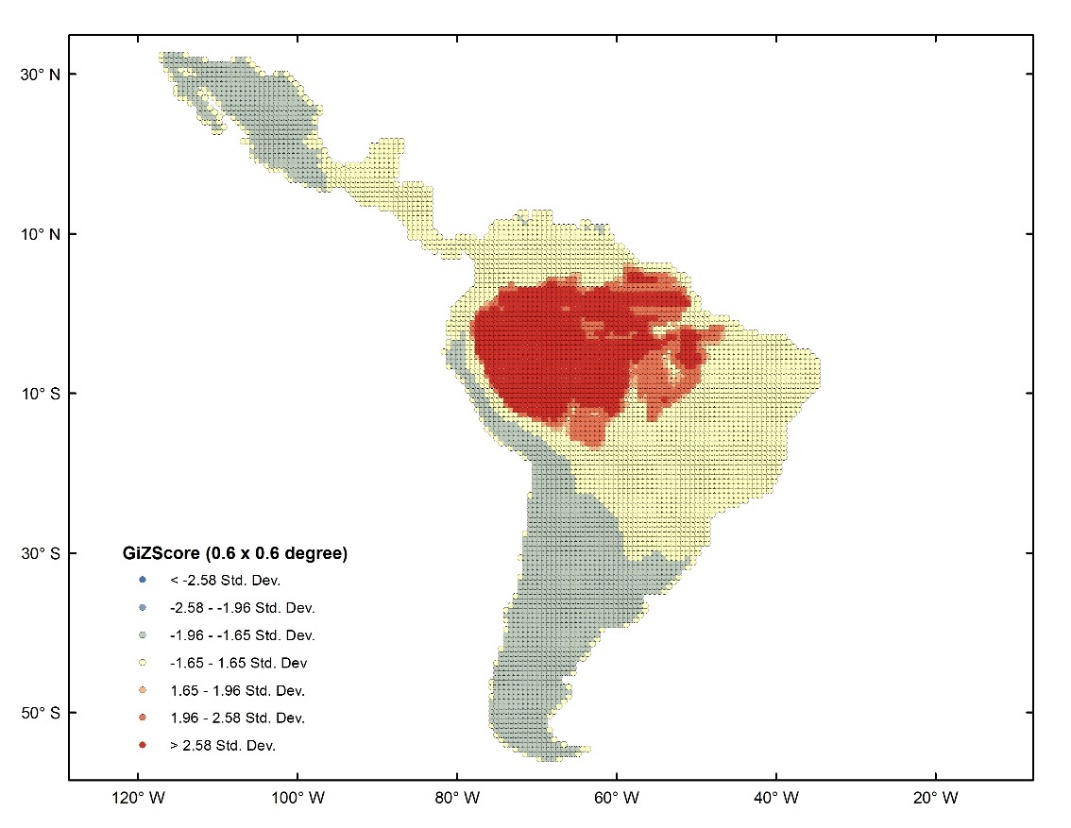
**

**Figure 12.** Biodiversity Hotspots defined using the Gi* statistics (Getis & Ord, 1992) to 0.6°.

**
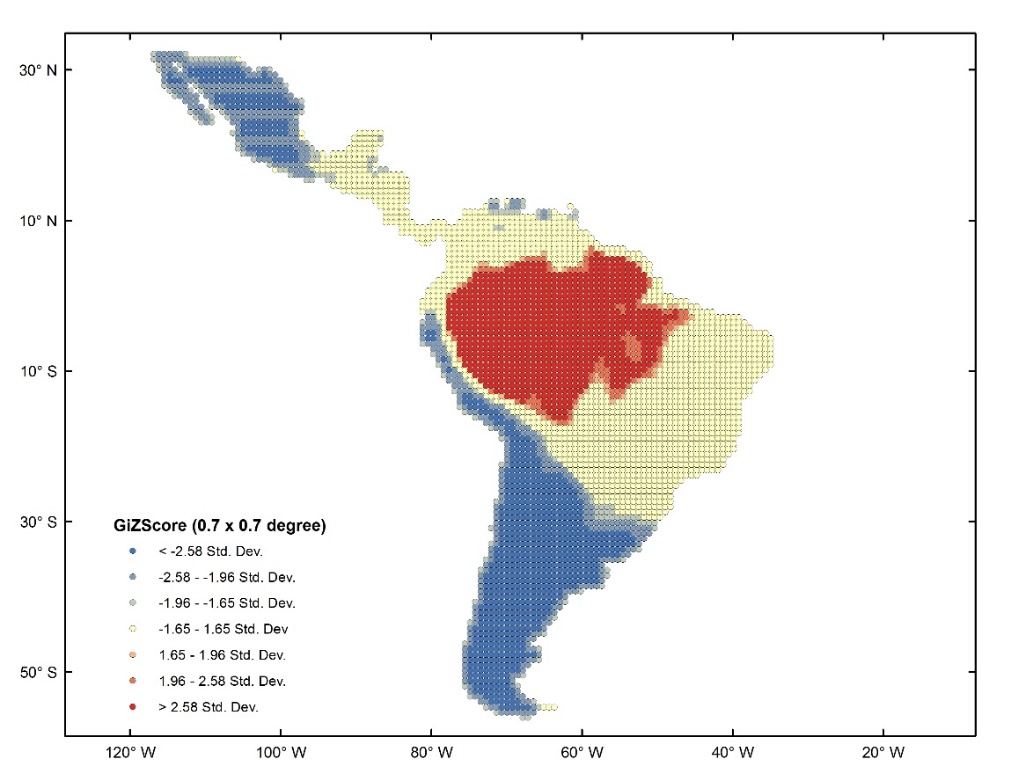
**

**Figure 13.** Biodiversity Hotspots defined using the Gi* statistics (Getis & Ord, 1992) to 0.7°.


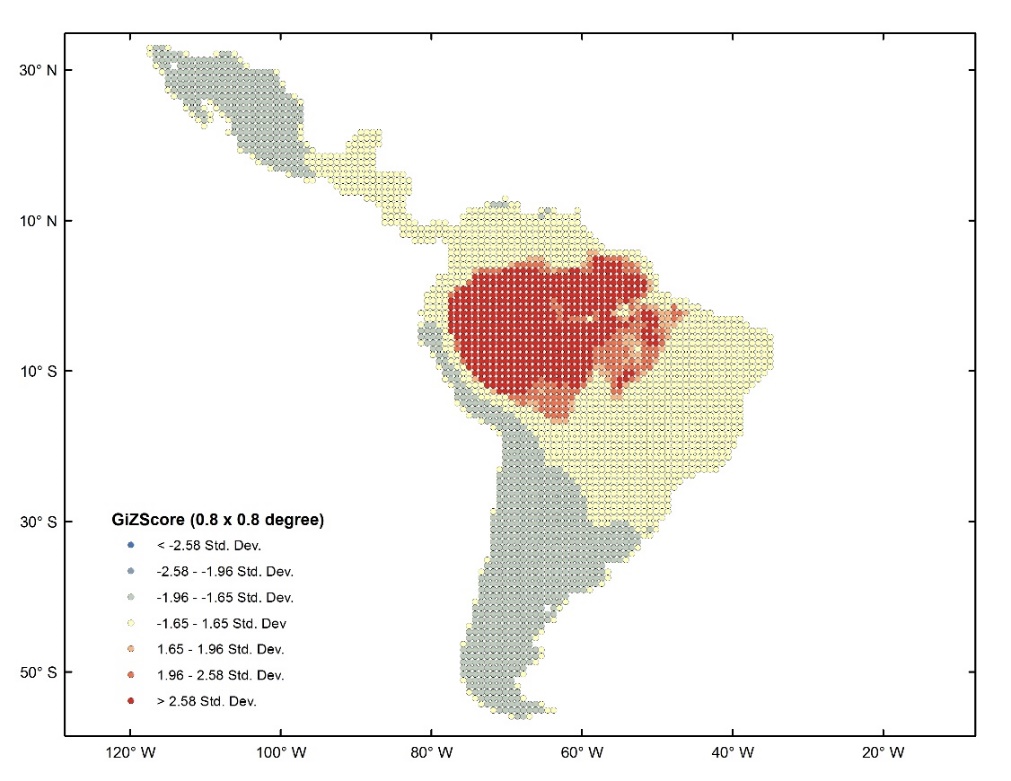


**Figure 14.** Biodiversity Hotspots defined using the Gi* statistics (Getis & Ord, 1992) to 0.8°.


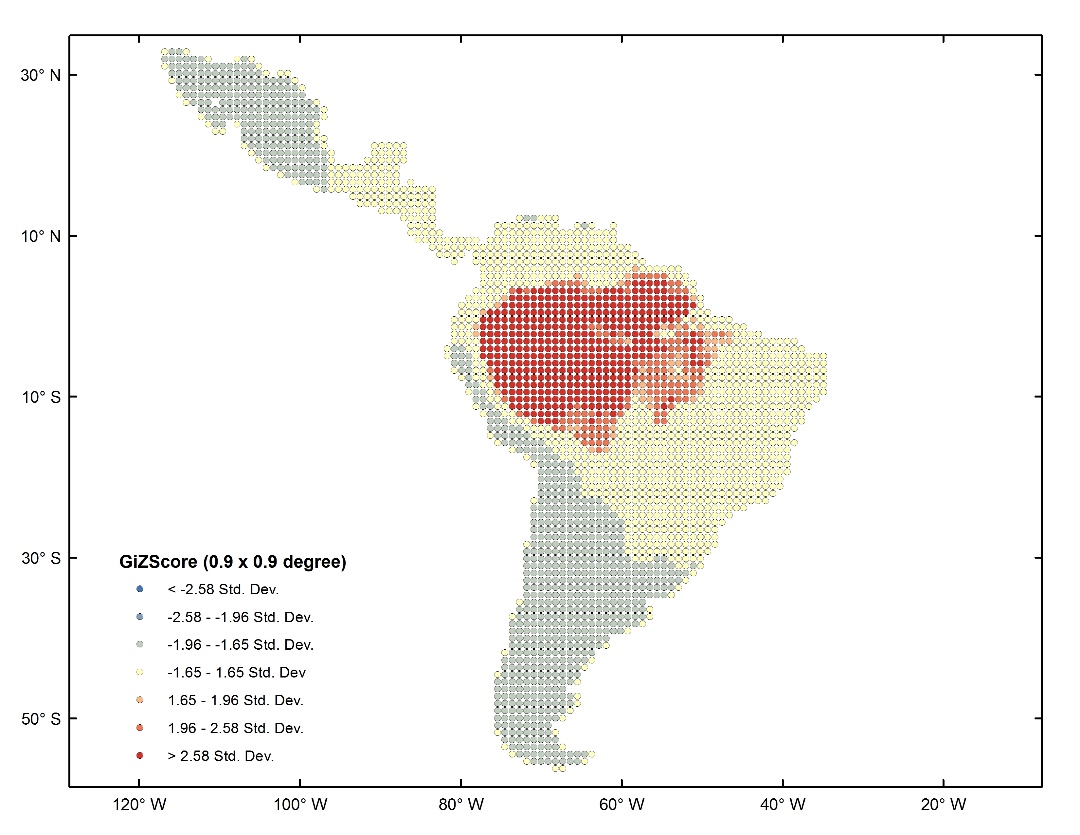


**Figure 15.** Biodiversity Hotspots defined using the Gi* statistics (Getis & Ord, 1992) to 0.9°.
