# Supplementary material for: Historical dynamics and current environmental effects explain the spatial distribution of species richness patterns of New World monkeys
Source: PeerJ. 2017 Sep 26;5:e3850. doi: 10.7717/peerj.3850 (PMC5621511; doi:10.7717/peerj.3850)
Supplement: Supplemental Information 2 — (A) Biogeographic stochastic mapping (BSM) in BioGeoBEARS package; (B) Ancestral area reconstruction in BioBeoBEARS package; (C) Quantile Regressions in Quantreg package. [file peerj-05-3850-s002.docx]

**SUPPLEMENTARY MATERIALS**

**Supplementary material 2: R scripts**

**A)**

**#BioGeoBEARS script 🡪 Biogeographic stochastic mapping (BSM)**

#Load required libraries

library(optimx)

library(FD)

library(snow)

library(parallel)

library(BioGeoBEARS)

source("http://phylo.wdfiles.com/local--files/biogeobears/cladoRcpp.R") # (needed now that traits model added; source FIRST!)

source("http://phylo.wdfiles.com/local--files/biogeobears/BioGeoBEARS_add_fossils_randomly_v1.R")

source("http://phylo.wdfiles.com/local--files/biogeobears/BioGeoBEARS_basics_v1.R")

source("http://phylo.wdfiles.com/local--files/biogeobears/BioGeoBEARS_calc_transition_matrices_v1.R")

source("http://phylo.wdfiles.com/local--files/biogeobears/BioGeoBEARS_classes_v1.R")

source("http://phylo.wdfiles.com/local--files/biogeobears/BioGeoBEARS_detection_v1.R")

source("http://phylo.wdfiles.com/local--files/biogeobears/BioGeoBEARS_DNA_cladogenesis_sim_v1.R")

source("http://phylo.wdfiles.com/local--files/biogeobears/BioGeoBEARS_extract_Qmat_COOmat_v1.R")

source("http://phylo.wdfiles.com/local--files/biogeobears/BioGeoBEARS_generics_v1.R")

source("http://phylo.wdfiles.com/local--files/biogeobears/BioGeoBEARS_models_v1.R")

source("http://phylo.wdfiles.com/local--files/biogeobears/BioGeoBEARS_on_multiple_trees_v1.R")

source("http://phylo.wdfiles.com/local--files/biogeobears/BioGeoBEARS_plots_v1.R")

source("http://phylo.wdfiles.com/local--files/biogeobears/BioGeoBEARS_readwrite_v1.R")

source("http://phylo.wdfiles.com/local--files/biogeobears/BioGeoBEARS_simulate_v1.R")

source("http://phylo.wdfiles.com/local--files/biogeobears/BioGeoBEARS_SSEsim_makePlots_v1.R")

source("http://phylo.wdfiles.com/local--files/biogeobears/BioGeoBEARS_SSEsim_v1.R")

source("http://phylo.wdfiles.com/local--files/biogeobears/BioGeoBEARS_stochastic_mapping_v1.R")

source("http://phylo.wdfiles.com/local--files/biogeobears/BioGeoBEARS_stratified_v1.R")

source("http://phylo.wdfiles.com/local--files/biogeobears/BioGeoBEARS_univ_model_v1.R")

source("http://phylo.wdfiles.com/local--files/biogeobears/calc_uppass_probs_v1.R")

source("http://phylo.wdfiles.com/local--files/biogeobears/calc_loglike_sp_v01.R")

source("http://phylo.wdfiles.com/local--files/biogeobears/get_stratified_subbranch_top_downpass_likelihoods_v1.R")

source("http://phylo.wdfiles.com/local--files/biogeobears/runBSM_v1.R")

source("http://phylo.wdfiles.com/local--files/biogeobears/stochastic_map_given_inputs.R")

source("http://phylo.wdfiles.com/local--files/biogeobears/summarize_BSM_tables_v1.R")

source("http://phylo.wdfiles.com/local--files/biogeobears/BioGeoBEARS_traits_v1.R") # added traits model

calc_loglike_sp = compiler::cmpfun(calc_loglike_sp_prebyte) # crucial to fix bug in uppass calculations

calc_independent_likelihoods_on_each_branch = compiler::cmpfun(calc_independent_likelihoods_on_each_branch_prebyte)

##based on the BiogeoBEARS object that contain the best-supported BioGeoBEARS model

model_name = "DEC"

# res = resDEC

clado_events_tables = NULL

ana_events_tables = NULL

lnum = 0

BSM_inputs_fn = "BSM_inputs_file.Rdata"

runInputsSlow = TRUE

if (runInputsSlow)

{

stochastic_mapping_inputs_list = get_inputs_for_stochastic_mapping(res=res)

save(stochastic_mapping_inputs_list, file=BSM_inputs_fn)

} else {

# Loads to "stochastic_mapping_inputs_list"

load(BSM_inputs_fn)

} # END if (runInputsSlow)

names(stochastic_mapping_inputs_list)

stochastic_mapping_inputs_list$phy2

stochastic_mapping_inputs_list$COO_weights_columnar

stochastic_mapping_inputs_list$unconstr

set.seed(seed=as.numeric(Sys.time()))

runBSMslow = TRUE

if (runBSMslow == TRUE)

{

# Saves to: RES_clado_events_tables.Rdata

# Saves to: RES_ana_events_tables.Rdata

BSM_output = runBSM(res, stochastic_mapping_inputs_list=stochastic_mapping_inputs_list, maxnum_maps_to_try=100, nummaps_goal=1000, maxtries_per_branch=40000, save_after_every_try=TRUE, savedir=getwd(), seedval=12345, wait_before_save=0.01)

RES_clado_events_tables = BSM_output$RES_clado_events_tables

RES_ana_events_tables = BSM_output$RES_ana_events_tables

} else {

# Load previously saved...

# Loads to: RES_clado_events_tables

load(file="RES_clado_events_tables.Rdata")

# Loads to: RES_ana_events_tables

load(file="RES_ana_events_tables.Rdata")

BSM_output = NULL

BSM_output$RES_clado_events_tables = RES_clado_events_tables

BSM_output$RES_ana_events_tables = RES_ana_events_tables

} # END if (runBSMslow == TRUE)

clado_events_tables = BSM_output$RES_clado_events_tables

ana_events_tables = BSM_output$RES_ana_events_tables

head(clado_events_tables[[1]])

head(ana_events_tables[[1]])

length(clado_events_tables)

length(ana_events_tables)

head(clado_events_tables[[1]])

head(ana_events_tables[[1]])

length(clado_events_tables)

length(ana_events_tables)

length(clado_events_tables)

length(ana_events_tables)

head(clado_events_tables[[1]][,-20])

tail(clado_events_tables[[1]][,-20])

head(ana_events_tables[[1]])

tail(ana_events_tables[[1]])

areanames = names(tipranges@df)

actual_names = areanames

actual_names

dmat_times = get_dmat_times_from_res(res=res, numstates=NULL)

dmat_times

clado_events_tables = BSM_output$RES_clado_events_tables

ana_events_tables = BSM_output$RES_ana_events_tables

BSMs_w_sourceAreas = simulate_source_areas_ana_clado(res, clado_events_tables, ana_events_tables, areanames)

clado_events_tables = BSMs_w_sourceAreas$clado_events_tables

ana_events_tables = BSMs_w_sourceAreas$ana_events_tables

counts_list = count_ana_clado_events(clado_events_tables, ana_events_tables, areanames, actual_names)

hist_event_counts(counts_list, pdffn=paste0(model_name, "_histograms_of_event_counts.pdf"))

**B)**

**#BioGeoBEARS script🡪 Ancestral area reconstruction**

#Load required libraries

library(optimx)

library(FD)

library(snow)

library(parallel)

library(BioGeoBEARS)

source("http://phylo.wdfiles.com/local--files/biogeobears/cladoRcpp.R") # (needed now that traits model added; source FIRST!)

source("http://phylo.wdfiles.com/local--files/biogeobears/BioGeoBEARS_add_fossils_randomly_v1.R")

source("http://phylo.wdfiles.com/local--files/biogeobears/BioGeoBEARS_basics_v1.R")

source("http://phylo.wdfiles.com/local--files/biogeobears/BioGeoBEARS_calc_transition_matrices_v1.R")

source("http://phylo.wdfiles.com/local--files/biogeobears/BioGeoBEARS_classes_v1.R")

source("http://phylo.wdfiles.com/local--files/biogeobears/BioGeoBEARS_detection_v1.R")

source("http://phylo.wdfiles.com/local--files/biogeobears/BioGeoBEARS_DNA_cladogenesis_sim_v1.R")

source("http://phylo.wdfiles.com/local--files/biogeobears/BioGeoBEARS_extract_Qmat_COOmat_v1.R")

source("http://phylo.wdfiles.com/local--files/biogeobears/BioGeoBEARS_generics_v1.R")

source("http://phylo.wdfiles.com/local--files/biogeobears/BioGeoBEARS_models_v1.R")

source("http://phylo.wdfiles.com/local--files/biogeobears/BioGeoBEARS_on_multiple_trees_v1.R")

source("http://phylo.wdfiles.com/local--files/biogeobears/BioGeoBEARS_plots_v1.R")

source("http://phylo.wdfiles.com/local--files/biogeobears/BioGeoBEARS_readwrite_v1.R")

source("http://phylo.wdfiles.com/local--files/biogeobears/BioGeoBEARS_simulate_v1.R")

source("http://phylo.wdfiles.com/local--files/biogeobears/BioGeoBEARS_SSEsim_makePlots_v1.R")

source("http://phylo.wdfiles.com/local--files/biogeobears/BioGeoBEARS_SSEsim_v1.R")

source("http://phylo.wdfiles.com/local--files/biogeobears/BioGeoBEARS_stochastic_mapping_v1.R")

source("http://phylo.wdfiles.com/local--files/biogeobears/BioGeoBEARS_stratified_v1.R")

source("http://phylo.wdfiles.com/local--files/biogeobears/BioGeoBEARS_univ_model_v1.R")

source("http://phylo.wdfiles.com/local--files/biogeobears/calc_uppass_probs_v1.R")

source("http://phylo.wdfiles.com/local--files/biogeobears/calc_loglike_sp_v01.R")

source("http://phylo.wdfiles.com/local--files/biogeobears/get_stratified_subbranch_top_downpass_likelihoods_v1.R")

source("http://phylo.wdfiles.com/local--files/biogeobears/runBSM_v1.R")

source("http://phylo.wdfiles.com/local--files/biogeobears/stochastic_map_given_inputs.R")

source("http://phylo.wdfiles.com/local--files/biogeobears/summarize_BSM_tables_v1.R")

source("http://phylo.wdfiles.com/local--files/biogeobears/BioGeoBEARS_traits_v1.R") # added traits model

calc_loglike_sp = compiler::cmpfun(calc_loglike_sp_prebyte) # crucial to fix bug in uppass calculations

calc_independent_likelihoods_on_each_branch = compiler::cmpfun(calc_independent_likelihoods_on_each_branch_prebyte)

#setting the working directory

extdata_dir = np(system.file("extdata", package="BioGeoBEARS"))

extdata_dir

list.files(extdata_dir)

#read files (phylogenetic dated tree and area data)

trfn = np(paste(addslash(extdata_dir), "Platyrrhini.nwk", sep=""))

moref(trfn)

tr = read.tree(trfn)

geogfn = np(paste(addslash(extdata_dir), "Platyrrhini_area.data", sep=""))

tipranges = getranges_from_LagrangePHYLIP(lgdata_fn=geogfn)

#setting BiogeoBEARS object

max_range_size = 3

BioGeoBEARS_run_object = define_BioGeoBEARS_run()

BioGeoBEARS_run_object$trfn = trfn

BioGeoBEARS_run_object$geogfn = geogfn

BioGeoBEARS_run_object$max_range_size = max_range_size

BioGeoBEARS_run_object$min_branchlength = 0.000001

BioGeoBEARS_run_object$include_null_range = TRUE

BioGeoBEARS_run_object$speedup = TRUE

BioGeoBEARS_run_object$use_optimx = TRUE

BioGeoBEARS_run_object$num_cores_to_use = 3

BioGeoBEARS_run_object$force_sparse = FALSE

BioGeoBEARS_run_object$return_condlikes_table = TRUE

BioGeoBEARS_run_object$calc_TTL_loglike_from_condlikes_table = TRUE

BioGeoBEARS_run_object$calc_ancprobs = TRUE

BioGeoBEARS_run_object

check_BioGeoBEARS_run(BioGeoBEARS_run_object)

# run DEC model

runslow = TRUE

resfn = "Platyrrhini_DEC.Rdata"

if (runslow)

{

res = bears_optim_run(BioGeoBEARS_run_object)

res

save(res, file=resfn)

resDEC = res

} else {

# Loads to "res"

load(resfn)

resDEC = res

}

### Run DEC+J

# setting BiogeoBEARS object

BioGeoBEARS_run_object = define_BioGeoBEARS_run()

BioGeoBEARS_run_object$trfn = trfn

BioGeoBEARS_run_object$geogfn = geogfn

BioGeoBEARS_run_object$max_range_size = max_range_size

BioGeoBEARS_run_object$min_branchlength = 0.000001

BioGeoBEARS_run_object$include_null_range = TRUE

BioGeoBEARS_run_object$speedup = TRUE

BioGeoBEARS_run_object$use_optimx = TRUE

BioGeoBEARS_run_object$num_cores_to_use = 1

BioGeoBEARS_run_object$force_sparse = FALSE

BioGeoBEARS_run_object = readfiles_BioGeoBEARS_run(BioGeoBEARS_run_object)

# Good default settings to get ancestral states

BioGeoBEARS_run_object$return_condlikes_table = TRUE

BioGeoBEARS_run_object$calc_TTL_loglike_from_condlikes_table = TRUE

BioGeoBEARS_run_object$calc_ancprobs = TRUE

# Set up DEC+J model (Adding the parameter "j")

dstart = resDEC$outputs@params_table["d","est"]

estart = resDEC$outputs@params_table["e","est"]

jstart = 0.0001

# Input starting values for d, e

BioGeoBEARS_run_object$BioGeoBEARS_model_object@params_table["d","init"] = dstart

BioGeoBEARS_run_object$BioGeoBEARS_model_object@params_table["d","est"] = dstart

BioGeoBEARS_run_object$BioGeoBEARS_model_object@params_table["e","init"] = estart

BioGeoBEARS_run_object$BioGeoBEARS_model_object@params_table["e","est"] = estart

# Add j as a free parameter

BioGeoBEARS_run_object$BioGeoBEARS_model_object@params_table["j","type"] = "free"

BioGeoBEARS_run_object$BioGeoBEARS_model_object@params_table["j","init"] = jstart

BioGeoBEARS_run_object$BioGeoBEARS_model_object@params_table["j","est"] = jstart

check_BioGeoBEARS_run(BioGeoBEARS_run_object)

#run DEC+j model

resfn = "Platyrrhini_DEC+J.Rdata"

runslow = TRUE

if (runslow)

{

#sourceall("/Dropbox/_njm/__packages/BioGeoBEARS_setup/")

res = bears_optim_run(BioGeoBEARS_run_object)

res

save(res, file=resfn)

resDECj = res

} else {

# Loads to "res"

load(resfn)

resDECj = res

}

## Model comparison

LnL_2 = get_LnL_from_BioGeoBEARS_results_object(resDEC)

LnL_1 = get_LnL_from_BioGeoBEARS_results_object(resDECj)

numparams1 = 3

numparams2 = 2

stats = AICstats_2models(LnL_1, LnL_2, numparams1, numparams2)

stats

# DEC, null model for Likelihood Ratio Test (LRT)

res2 = extract_params_from_BioGeoBEARS_results_object(results_object=resDEC, returnwhat="table", addl_params=c("j"), paramsstr_digits=4)

# DEC+J, alternative model for Likelihood Ratio Test (LRT)

res1 = extract_params_from_BioGeoBEARS_results_object(results_object=resDECj, returnwhat="table", addl_params=c("j"), paramsstr_digits=4)

rbind(res2, res1)

tmp_tests = conditional_format_table(stats)

restable = rbind(restable, res2, res1)

teststable = rbind(teststable, tmp_tests)

##plot_DEC (best supported model)

analysis_titletxt ="BioGeoBEARS DEC on Platyrrhini"

# Setup

results_object = resDEC

scriptdir = np(system.file("extdata/a_scripts", package="BioGeoBEARS"))

# States

res2 = plot_BioGeoBEARS_results(results_object, analysis_titletxt, addl_params=list("j"), plotwhat="text", label.offset=0.45, tipcex=0.7, statecex=0.7, splitcex=0.6, titlecex=0.8, plotsplits=FALSE, cornercoords_loc=scriptdir, include_null_range=TRUE, tr=tr, tipranges=tipranges)

# Pie chart

plot_BioGeoBEARS_results(results_object, analysis_titletxt, addl_params=list("j"), plotwhat="pie", label.offset=0.45, tipcex=0.7, statecex=0.7, splitcex=0.6, titlecex=0.8, plotsplits=FALSE, cornercoords_loc=scriptdir, include_null_range=TRUE, tr=tr, tipranges=tipranges)

**C)**

**Quantile regression script**

#load package quantreg: quantile regression#

library(quantreg)

data<-read.csv("data.csv", header=T,sep=";", dec=".")

attach(data)

#variables

y <- cbind(richness)

x <- cbind(aet, bio2, bio3, bio5, bio7, bio11, bio12, bio13, bio16, bio17, bio19, pet, densidad)

summary(x)

summary(y)

#OLS

regression<- lm(y ~ x, data= data)

summary(regression)

#quantile regression

quantreg25<- rq(y ~ x, data= data, tau=0.25)

summary(quantreg25)

quantreg50<- rq(y ~ x, data= data, tau=0.5)

summary(quantreg50)

quantreg75<- rq(y ~ x, data= data, tau=0.75)

summary(quantreg75)

#Regression by quantiles simultaneously###

quantreg2575<- rq(y ~ x, data= data, tau=c(0.25, 0.75))

summary(quantreg2575)

#Anova###

anova(quantreg25, quantreg75)

#plotting data

quantreg.all<- rq(y ~ x, tau=seq(0.05, 0.95, by= 0.05))

quantreg.plot<-summary(quantreg.all)

plot(quantreg.plot)

quantreg.all<- rq(y ~ x, tau=seq(0.01, 0.99, by= 0.01))

summary(quantreg.all)

qt0.01<-rq(y~x,tau=0.01)

summary(qt0.01,se="boot",R=1000)

qt0.99<-rq(y~x,tau=0.99)

summary(qt0.99,se="boot",R=1000)

plot((y~x),pch=16,cex=0.7, ylab="Richness",cex.lab=1.5,cex.axis=1,xlab="AET")

abline(qt0.01,lwd=2, col=2)

abline(qt0.99,lwd=2, col=2)
